# Recombinant *Pseudomonas* growing on non-natural fluorinated substrates shows stress but overall tolerance to cytoplasmically released fluoride anion

**DOI:** 10.1128/mbio.02785-23

**Published:** 2023-12-08

**Authors:** Anthony G. Dodge, Calvin J. Thoma, Madeline R. O’Connor, Lawrence P. Wackett

**Affiliations:** 1 Department of Biochemistry, Molecular Biology and Biophysics and Biotechnology Institute, University of Minnesota, Twin Cities, Minnesota, USA; University of Washington School of Medicine, Seattle, Washington, USA

**Keywords:** fluoride, organofluorine, PFAS, bacterium, *Pseudomonas*, stress, biodegradation, defluorination, enzyme, *Delftia*, α-fluorophenylacetic acid, 2-fluoropropionic acid

## Abstract

Microbial response to toxic fluoride anion has traditionally been studied by adding inorganic fluoride salts to growth media. Fluoride is known to spontaneously transit the membrane as hydrogen fluoride (HF) and manifests significant toxicity in the cytoplasm. The present study investigated how microbes respond to high levels of HF generated directly in the cytoplasm to better understand potential limits of microbial defluorination of organofluorine compounds. For that purpose, *Pseudomonas putida* ATCC 12633 was engineered to express a defluorinase enzyme from *Delftia acidovorans* strain B that had high activity in producing growth-supporting alcohols from organofluorinated compounds of xenobiotic origin. Genome annotation predicted the growth of *P. putida* ATCC 12633 on all possible chiral products from 2-fluoropropionic acid and α-fluorophenylacetic acid when defluroinases are expressed. The defluorinase was shown to have complete enantioselectivity for (*S*)-fluoro substrates via experimental and computational modeling methods. The bacterium grew to a high turbidity with stoichiometric release of fluoride from the (*S*)-enantiomers into the medium. The highest yield of fluoride obtained was 50 mM with 2-fluoropropionic acid as the growth substrate. The growth yield was significantly lowered by 41% with α-fluorophenylacetic acid or 2-fluoropropionic acid compared to (*S*)-mandelic acid or D-lactic acid, respectively. Fluoride stress was also indicated by longer lag phases, slower growth, and cell morphology changes on fluorinated substrates or the cognate alcohols with NaF in the medium. In total, these studies show the potential for engineering bacterial defluorination of non-natural substrates within limits posed by fluoride stress.

**IMPORTANCE:**

Society uses thousands of organofluorine compounds, sometimes denoted per- and polyfluoroalkyl substances (PFAS), in hundreds of products, but recent studies have shown some to manifest human and environmental health effects. As a class, they are recalcitrant to biodegradation, partly due to the paucity of fluorinated natural products to which microbes have been exposed. Another limit to PFAS biodegradation is the intracellular toxicity of fluoride anion generated from C-F bond cleavage. The present study identified a broader substrate specificity in an enzyme originally studied for its activity on the natural product fluoroacetate. A recombinant *Pseudomonas* expressing this enzyme was used here as a model system to better understand the limits and effects of a high level of intracellular fluoride generation. A fluoride stress response has evolved in bacteria and has been described in *Pseudomonas* spp. The present study is highly relevant to organofluorine compound degradation or engineered biosynthesis in which fluoride anion is a substrate.

## INTRODUCTION

Fluorinated pesticides, lubricants, and pharmaceuticals are increasingly being produced for use in more than 200 applications (1, 2). Due to health and environmental concerns, fluorinated compounds (FCs) are increasingly under scrutiny and regulation (1, 2). A major concern is the poor biodegradability of FCs. The observation of very limited microbial FC degradation in the environment has been discussed as arising potentially from several factors: (i) a limited set of enzymes cleaving C-F bonds due to the largely non-natural origin of FCs, (ii) the inherent low chemical reactivity of the C-F bond of FCs, (iii) a disruption of metabolic and physiological processes by some FCs, and (iv) if C-F bonds are cleaved in the cytoplasm, fluoride anion is generated intracellularly, where it is known to cause toxic effects at low concentrations (2–7). This study focused on the latter issue.

Fluoride toxicity has largely been studied by adding inorganic fluoride, typically NaF, into the growth medium, which mimics microbial exposure to fluoride-containing minerals in the environment (6, 8, 9). Extracellular fluoride enters cells mainly as hydrogen fluoride (HF) that dissociates to fluoride anion at the pH maintained inside bacterial cells. As little as 0.1 mM intracellular fluoride is thought to impart significant toxicity due to binding to metals in essential enzymes such as enolase, pyrophosphatase, and ATPases (5, 10, 11). This toxicity underlies part of the utility of fluoride in dental products that reduce caries.

Some bacteria are more tolerant to fluoride and can cope with fluoride anion in multiple ways. The principal mechanism is to export the fluoride from the cell. Two distinct types of exporters have been identified—fluoride channels produced by *crcB* gene products and a fluoride/proton antiporter that resembles chloride transporters, known as CLC^F^ antiporters (9, 12, 13). Some fluoride transporters are controlled by a fluoride-sensitive riboswitch that also upregulates numerous other genes, many of undefined functions (8). It is largely unknown how bacteria will be able to maintain the biodegradation of very high levels of organofluorine compounds via intracellular enzymes in which all of the fluoride anion is released directly into the cytoplasm. Because of the high electronegativity of fluorine, all mechanisms of C-F bond cleavage make fluoride anion as a product (14).

Most biodegradation studies of bacteria growing on organofluorine compounds occur via oxygenative, reductive, and hydrolytic C-F bond cleavage, based on the organic products formed (15–18). All of those reactions are thought to occur intracellularly. In those cases where fluoride was monitored in the growth medium, the levels typically fall in the range of 1–10 mM (19–22). The potential for toxicity from fluoride anion was not addressed in those studies.

There are at present only a limited number of known enzymes that cleave C-F bonds, with fluoroacetate dehalogenase (FAcD) being the most well-studied (15, 23–26). FAcD catalyzes an overall hydrolytic cleavage of the C-F bond of fluoroacetate, thought to be the most prevalent of a rare set of fluorinated natural products (26, 27). X-ray structure and mutagenesis studies have illustrated how the FAcD from *Rhodopseudomonas palustris* ATCC BAA-98 activates fluoroacetate to cleave the C-F bond. However, the enzyme is relatively substrate-specific. For the present study, we sought a similar type of hydrolytic defluorinase to generate growth-supporting alcohols and a high intracellular flux of fluoride anion.

The present study consisted of several steps: (i) identify a highly active defluorinating enzyme, (ii) express the enzyme in a suitable host to grow on the products of the defluorinase, (iii) examine the effects of intracellular fluoride release, and (iv) determine what level of fluoride anion is ultimately inhibitory and toxic under these conditions. A soluble hydrolytic defluorinase from *Delftia acidovorans* (formerly *Moraxella* sp.) strain B (28) and the well-studied FAcD from *R. palustris* ATCC BAA-98 (15) were purified and compared, and the former was shown to be more suitable due to higher reaction rates and broader substrate specificity. The *Delftia* enzyme defluorinated α-fluorophenylacetic acid and 2-fluoropropionic acid, allowing us to avoid the use of fluoroacetate, which is itself highly toxic. The two less toxic substrates were shown to be stereoselectively defluorinated to produce enantiospecific alcohols that served as growth substrates for *Pseudomonas putida* ATCC 12633. Strain 12663 cells expressing the cytoplasmic *Delftia* defluorinase recombinantly grew on the fluorinated compounds and reached fluoride levels in the medium up to 50 mM. Fluoride stress due to cytoplasmic fluoride release was demonstrated and characterized. These studies suggested that bacteria with robust fluoride anion resistance functions will have the capacity to carry out extensive degradation of fluorinated compounds.

## RESULTS

### Purification and comparison of defluorinases to use for *in vivo* studies

We sought to create high levels of intracellular fluoride release in an engineered bacterium to investigate whether a high flux of intracellular fluoride would toxify the bacterium and shut down metabolism. The goal was to promote the use of fluorinated organic compounds as the sole source of carbon. The ideal enzyme sought was a hydrolytic dehalogenase that would react rapidly with substrates other than fluoroacetate, since fluoroacetate is highly toxic. A hydrolytic defluorination reaction does not require energy input, allowing a direct comparison between growth on a fluorinated substrate and its cognate product.

Initially, two hydrolytic enzymes were tested for this purpose. One was the well-studied *R. palustris* FAcD, and the second was a less-studied defluorinase from *D. acidovorans* strain B (28). Both enzymes were purified to homogeneity (Fig. S1). Each enzyme was independently tested against a range of fluorinated compounds, measuring rates of fluoride release (Table 1). The enzymes were assayed at both pH 7.5 and pH 9.0. The higher pH was also included because it was the pH optimum for both defluorinases and allowed comparison to other studies (15, 24, 28).

**TABLE 1:** Specific activity of purified enzymes with fluorinated substrates assayed as described in Materials and Methods^*c*^

Substrate	*R. palustris* FAcD^*a*^ (µmol min^−1^ mg^−1^)	*D. acidovorans* defluorinase^*b*^ (µmol min^−1^ mg^−1^)
α-Fluorophenylacetic acid	2.5 ± 0.1	14.5 ± 0.9
2-Fluoropropionic acid	0.063 ± 0.002	1.7 ± 0.2
2-Chloro-2-fluoroacetic acid	<0.01	0.13 ± 0.01
α,α-Difluorophenylacetic acid	<0.01	<0.01
2,2-Difluoropropionic acid	<0.01	<0.01
2-Fluoroacrylic acid	<0.01	<0.01
Fluoroacetonitrile	<0.01	<0.01
Benzylfluoride	<0.01	<0.01

^*a*^ Overexpressed recombinantly in *Escherichia coli* BL21(DE3).

^*b*^ Overexpressed recombinantly in *P. putida* ATCC 12633.

^*c*^ The values represent the averages and standard deviations of triplicate reactions at three time points.

The *Delftia* enzyme showed an almost 6-fold higher rate of defluorination with α-fluorophenylacetic acid and a 27-fold higher rate with 2-fluoropropionic acid compared to the *R. palustris* FAcD. Neither enzyme was reactive with difluorinated carbon centers or with a fluoroolefin substrate. To achieve the goal of releasing high levels of intracellular fluoride, the *Delftia* enzyme was chosen for expression in a suitable host that would grow on either enantiomer of the defluorination products: mandelic acid and lactic acid. Other homologous enzymes had been demonstrated to show enantioselectivity, but this had not been determined directly for the *Delftia* enzyme.

### Selection of a metabolically compatible bacterium

**Fig 1:**
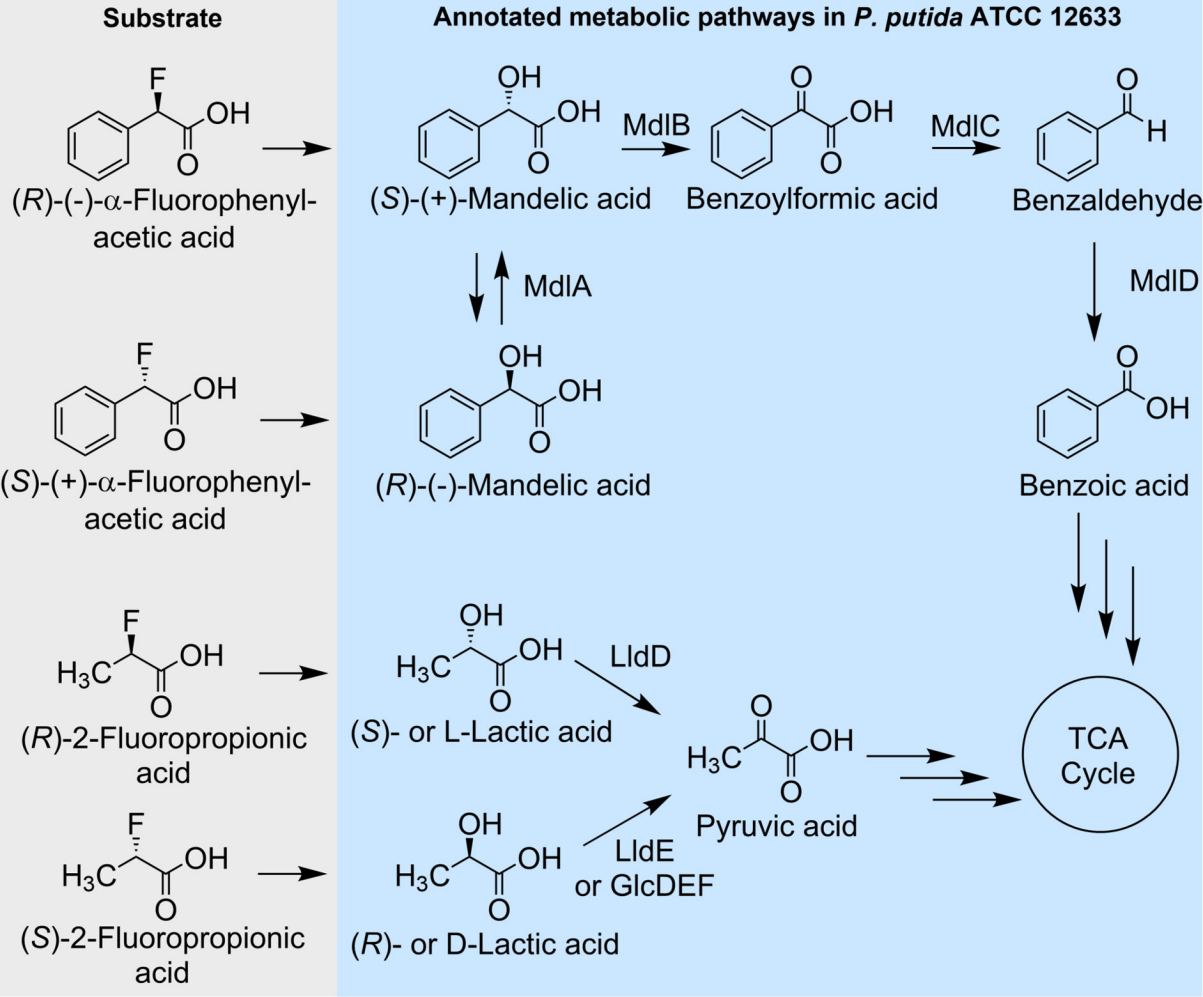
Potential growth pathways with fluorinated acids via annotated metabolic pathways determined for *P. putida* ATCC 12633. The stacked arrows leading from benzoic acid and pyruvic acid represent that multiple reaction steps are required to produce TCA cycle intermediates. The genes and enzymes involved in benzoate and pyruvate metabolism are highlighted in Table S1.

Based on the reactivity of the *Delftia* defluorinase demonstrated here, the defluorination of α-fluorophenylacetic acid, 2-fluoropropionic acid, and chlorofluoroacetic acid would yield the organic products mandelic acid, lactic acid, and glyoxylic acid, respectively. We observed no growth on chlorofluoroacetic acid, the slowest substrate, so we focused on the first two. The stereochemical selectivity of the *Delftia* defluorinase with these substrates was unknown, so the goal was to express the enzyme in a bacterium that would metabolize and grow on (*S*)- and (*R*)-mandelic acid and D- and L-lactic acid and have likely transporters for α-fluorophenylacetic acid and 2-fluoropropionic acid based on known growth on analogous non-fluorinated compounds. Based on an analysis of multiple genomes, the bacterium *Pseudomonas putida* ATCC 12633 was selected. The overall logic of the choice of the bacterium is shown in Fig. 1 and represented in the genome annotations compiled in Table S1.

The genome annotation for *P. putida* ATCC 12633 showed a cluster of genes (*mndBCD*) annotated for the catabolism of (*S*)-mandelate through benzoate and, in a different genomic location, genes encoding benzoate catabolism to generate TCA cycle intermediates via catechol *ortho*-cleavage (Table S1). Moreover, if the defluorinase was stereoselective, either (*R*)- or (*S*)-α-fluorophenylacetic acid could support growth. (*S*)-Mandelate and (*R*)-mandelate would be interconvertible by a mandelate racemase encoded by the product of the *mndA* gene found in the same cluster (Fig. 1). While we did not identify a putative lactate racemase, we did identify a gene cluster (*lldRPDE*) with genes encoding separate L- or D-lactate dehydrogenases (*lldD* or *lldE*, respectively) (29–31). Both translated genes were highly similar to authentic D- and L-lactate dehydrogenases demonstrated in other *Pseudomonas* strains (29–31). We also found another gene cluster (*glcCDEF*) encoding a transcriptional regulator and the three subunits of a second assimilatory D-lactate dehydrogenase (GlcDEF) previously described in *P. putida* KT2440 (29) (Table S1).

### Expression of recombinant *Delftia* defluorinase in *P. putida* 12633 allows growth on α-fluoro carboxylic acids

**Fig 2:**
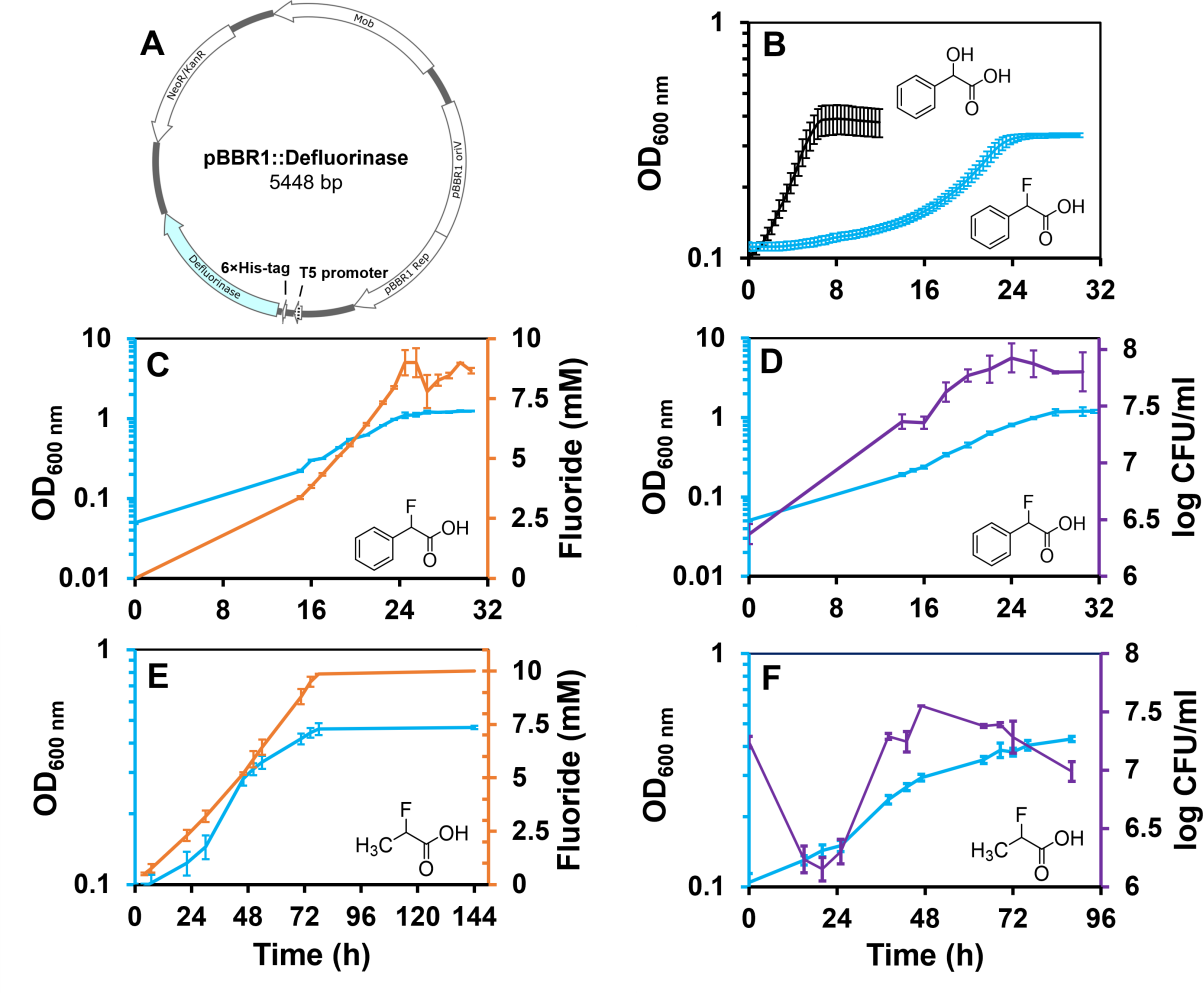
Engineering of *Pseudomonas putida* ATCC 12633 to use fluorinated substrates and determine fluoride release, increase in turbidity, optical density (OD_600 nm_), over time, and colony-forming units (CFUs) during the defluorination phase. The panels show (**A**) the recombinant plasmid containing the *Delftia* defluorinase gene, (**B**) a comparison of growth on mandelic acid and α-fluorophenylacetic acid with curves showing the averages and standard deviations of 10 separate determinations, (**C**) fluoride release during growth on α-fluorophenylacetic acid, (**D**) a comparison of OD_600_ and colony-forming units during growth on α-fluorophenylacetic acid, (**E**) fluoride release during growth on 2-fluoropropionic acid, and (**F**) a comparison of OD_600_ and CFU during growth on 2-fluoropropionic acid. In all cases, the substrates were supplied as the racemates and at a concentration of 20 mM, except for mandelic acid that was 10 mM. Panels C–F show averages and standard deviations from three replicates. Plasmid construction, cloning, optical density determinations, colony-forming units, and fluoride determinations were carried out as described in Materials and Methods.

Strain 12633 grew with racemic mandelic acid, (*S*)-mandelic acid, (*R*)-mandelic acid, racemic lactic acid, (L)-lactic acid, and (D)-lactic acid. A recombinant *P. putida* 12633 strain expressing the *Delftia* defluorinase constitutively (Fig. 2A) also grew on the same substrates. With both strains and all substrates, significant growth was observed within 24 h. The wild-type *P. putida* 12633 strain is not known to grow on fluorinated acids and showed no growth on our fluorinated test substrates here. Moreover, there were no identifiable native defluorinase genes observed in the genome sequence.

The recombinant *P. putida* 12633 expressing the *Delftia* defluorinase constitutively from a plasmid (Fig. 2A) was analyzed in growth studies with the non-natural compounds α-fluorophenylacetic acid or 2-fluoropropionic acid and the naturally occurring compound mandelic acid supplied as the sole carbon and energy source (Fig. 2B through F). The recombinant strain grew rapidly with mandelate, showing only a brief lag time of 1.2 h and a maximum growth rate of 0.23 h^−1^ as determined via a logarithmic fit using a QurvE computational tool (32) model (Fig. 2B). With the same conditions and model, growth on α-fluorophenylacetic acid showed a much longer lag time of 14.3 h and a lower maximum growth rate of 0.12 h.^−1^ A separate growth experiment on α-fluorophenylacetic acid determined fluoride release and revealed more than one-third of the substrate had been defluorinated (>3.3 mM) early in the growth phase, consistent with the defluorinase being constitutively expressed (Fig. 2C). This suggested that inhibition from fluoride might underlie the long lag phase. In another growth experiment, cell numbers estimated from turbidity (OD_600 nm_) were compared to cell counts by plating on rich media, to examine if fluoride toxicity might diminish viability (Fig. 2D). By this method, the cell numbers increased over the 24 h growth phase, indicating that the strain remained viable. When growth was tested with the slower defluorinase substrate, 2-fluoropropionic acid, the cells grew slowly for up to 30 h and subsequently grew more rapidly before plateauing after 72 h (Fig. 2E). Fluoride measurements showed a complete release of fluoride from one substrate enantiomer, indicating metabolizable substrate was depleted at 72 h. The significant release of fluoride during the slow growth phase up to 30 h was consistent with a likely toxic effect of fluoride. Fluoride toxicity was more strongly suggested by the colony-forming units on plating, which showed a 90% reduction in viable cells during the first 20 h, followed by a recovery phase during the period of 24–48 h that paralleled the period of faster growth.

Only half of the total fluorine atoms from 20 mM racemic fluorinated substrate were released in each case (Fig. 2C and E). This suggested that the *Delftia* defluorinase was largely or completely enantioselective. In light of that, we sought to determine the stereochemical course of the reactions by directly analyzing the reactions and modeling the enzyme.

### Substrate conversion and stereochemical outcomes

**Fig 3:**
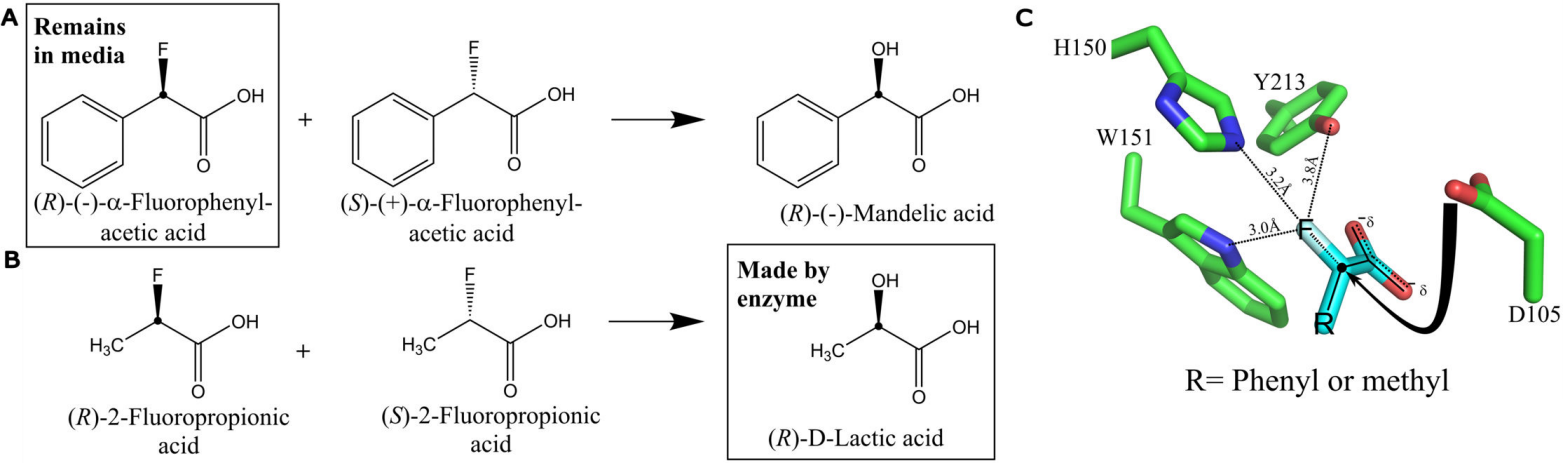
Determinations of defluorinase stereoselectivity. (**A**) Polarimetry was used to show that (***R***)-(−)-α-fluorophenylacetic acid remained in the growth medium following the transformation of racemic substrate by recombinant *P. putida* 12633 expressing the defluorinase. (**B**) Stereospecific lactic acid dehydrogenases were used to analyze the product of the defluorinase reacting with racemic 2-fluoropropionic acid. (**C**) Computational model of the defluorinase active site showing the proposed nucleophilic attack by D105 with inversion of configuration, converting (***S***)-substrates to (***R***)-products.

Since half of the racemic substrates remained and the other half was converted to alcohol products, the *Delftia* defluorinase was clearly stereoselective. The stereochemistry could be determined by examining the remaining substrate, analyzing the product, or modeling the active site and determining the stereochemical active site configuration catalyzing defluorination (Fig. 3). All those methods were utilized here. A growing cell culture was used to transform racemic α-fluorophenylacetic acid until the reaction was complete and 0.5 equivalents of the total fluorine were released as fluoride. The cells were removed, and the medium was extracted and concentrated as described in Materials and Methods.

The dried extracted material was quantified and dissolved in water. The specific rotation determined in a polarimeter was −110, compared to a reported specific rotation of −124 (33). This indicated that the material was largely, or totally, (*R*)-(−)-α-fluorophenylacetic acid (Fig. 3A). That led to the conclusion that (*S*)-(+)-α-fluorophenylacetic acid was the enantiomer transformed by the FAcD enzyme with the release of fluoride.

*In vitro*, the purified *Delftia* defluorinase enzyme was reacted with racemic 2-fluoropropionic acid until 0.5 equivalents of fluoride were released, at which point no more reaction was discernible. The reaction mixture was then further reacted with stereospecific D-lactate dehydrogenase and L-lactate dehydrogenase as measured by NADH formation. A significant rate of NADH formation was only observed with D-lactate dehydrogenase, indicating that (*S*)-2-fluoropropionic acid is selectively processed to (*R*)-lactic acid, more commonly referred to as D-lactic acid (Fig. 3B).

The results indicate that both substrates are processed with the inversion of configuration during the replacement of the fluorine atom with a hydroxyl group. This type of mechanism has been demonstrated with a defluorinase from *Burkholderia* for which an X-ray structure is available (34, 35). We used an AlphaFold (36, 37) model of the *Delftia* enzyme, for which an X-ray structure is not currently available, to infer if the defluorination reaction would also yield a product with inverted stereochemistry. The overall fold was similar to the *Burkholderia* enzyme. Substrates were docked into the enzyme as described in Materials and Methods. Substrate binding in the active site was constrained by the charged carboxylic acid and the R-group, a phenyl or methyl substituent (Fig. 3C). In this manner, attack of the aspartic acid residue would invert the configuration, converting (*S*)-oriented fluoro-substrates to (*R*)-configuration alcohols, after hydrolysis of the enzyme ester intermediate. The enzyme modeling is thus consistent with the direct stereochemical determinations. Moreover, the model showed a significant space for accommodating R-groups, consistent with reactivity with R = H, Cl, CH_3_ and phenyl ring substrates.

### Comparison of growth yields on α-fluoro and α-hydroxy acids

Based on the findings of the defluorinase stereoselectivity, comparative growth yields and times to reach the stationary phase were determined using fluorinated acids, their stereospecific cognate alcohol metabolites, and the alcohol metabolites with fluoride anion added. The carbon and energy available from the fluorinated and non-fluorinated substrates are equivalent, and the defluorination reaction is hydrolytic and energy-neutral. The comparison is designed to provide insights into the stress imposed by exogenously provided and internally generated fluoride anion.

The results highlight the differences in fluoride stress from exogenous media fluoride and fluoride released internally in comparison to no fluoride stress. With 10 mM fluoride added exogenously, the drop in growth yield was 22% (Table 2). With a level of α-fluorophenylacetic acid that released 10 mM fluoride internally, the decrease in growth yield was 41%. Moreover, the time to reach the stationary phase was only 9 h with sodium fluoride added and 29 h with α-fluorophenylacetic acid, due to a long lag phase (Table 2) in the case of internally generated fluoride.

**TABLE 2:** Comparative maximum cell density, fluoride release, and time for growth on fluorinated acids, their cognate alcohol metabolites, and the cognate metabolites with fluoride anion supplemented into the medium at the start of the culture^*a*^

Carbon source and fluoride added	Cell density (OD_600 nm_)	Fluoride released (mM)	Time to max OD_600 nm_ (h)
10 mM (*R*)-mandelic acid	2.07 ± 0.02	–	7
10 mM (*R*)-mandelic acid+10 mM NaF	1.61 ± 0.07	–	9
20 mM (*RS*)-α-fluorophenylacetic acid	1.22 ± 0.02	9.9 ± 0.1	29
10 mM (*R*)-(D)-lactic acid	0.627 ± 0.003	–	5
10 mM (*R*)-(D)-lactic acid+10 mM NaF	0.54 ± 0.01	–	8
20 mM (*RS*)-2-fluoropropionic acid	0.37 ± 0.01	9.9 ± 0.2	78

^*a*^ Values presented are the averages and standard deviations of three biological replicates. Similar results were obtained when the same experiment was repeated once independently.

Similar results were seen with lactic acid and fluoride compared to 2-fluoropropionic acid. In that case with fluoride in the medium, the growth yield was diminished by 14% compared to growth on lactic acid without fluoride. With 2-fluoropropionic acid, the growth yield was diminished by 41% compared to growth on D-lactic acid. Dramatically, the time to reach the stationary phase was 8 h with sodium fluoride in the medium and 78 h with 2-fluoropropionic acid. In a complementary experiment, we used α-chlorophenylacetic acid, which we found was also processed as a growth substrate (Fig. S2). That substrate did not show the substantial lag phase observed with α-fluorophenylacetic acid, consistent with the idea that the acidic substrate itself is not inhibitory, but rather the fluoride being released is mediating the growth delay.

In both cases, the reactive enantiomer of the fluorinated substrate was completely exhausted based on the yield of fluoride determined (Table 2). In total, the results show energy-demanding stress due to the cytoplasmic defluorination reaction, while also indicating a significant robustness of the recombinant *P. putida* strain to continue to process the fluorinated substrates to completion.

### Other indicators of fluoride stress

*P. putida* 12633 expressing the recombinant defluorinase was grown on minimal medium agar plates with either mandelic acid or α-fluorophenylacetic acid as the carbon source. The mandelic acid plates showed growth in 24 h, and relatively large, uniform-sized colonies were observed in 3 days. On α-fluorophenylacetic acid, no colonies were observable in the first few days, but growth was seen after 7 days.

Cells exposed to sodium fluoride and α-fluorophenylacetic acid in liquid media showed significant cell morphological changes. A 12.5 mM concentration of fluoride equivalents was provided from NaF or during the metabolism of 25 mM α-fluorophenylacetic acid. The cell differences were most pronounced during the mid-to-late exponential phase of growth (Fig. S3). With exposure to NaF, cells were discernibly shorter and narrower, with cell lengths decreasing from 4 microns to 2 microns (Fig. S3). Similar shortening and segmentation were observed in cells growing on α-fluorophenylacetic acid. Cells in the lag phase and beginning growth phase did not show very small cell types. This suggested that large cells were not shrinking but became shortened during cell division. A control set of experiments with sodium chloride did not show any distinct cell morphological changes with up to 50 mM of the salt, suggesting the observations are not due to salt stress and reflect a response to fluoride anion specifically.

In separate experiments, we tested cells for the accumulation of poly-hydroxyalkanoates, another potential indicator of stress in *Pseudomonas* strains (38). No significant difference was observed between cells grown on α-fluorophenylacetic acid or mandelic acid.

### Maximizing defluorination

As shown in Fig. 2C and E, fluoride levels in the medium are a direct measure of the amount of substrate consumed, and since the enzyme is intracellular, all of the fluoride anion is generated intracellularly. Increasing substrate levels will increase internal fluoride levels and increase fluoride stress. Here, we examined the response of recombinant *P. putida* 12633 to higher concentrations of fluorinated substrates.

**Fig 4:**
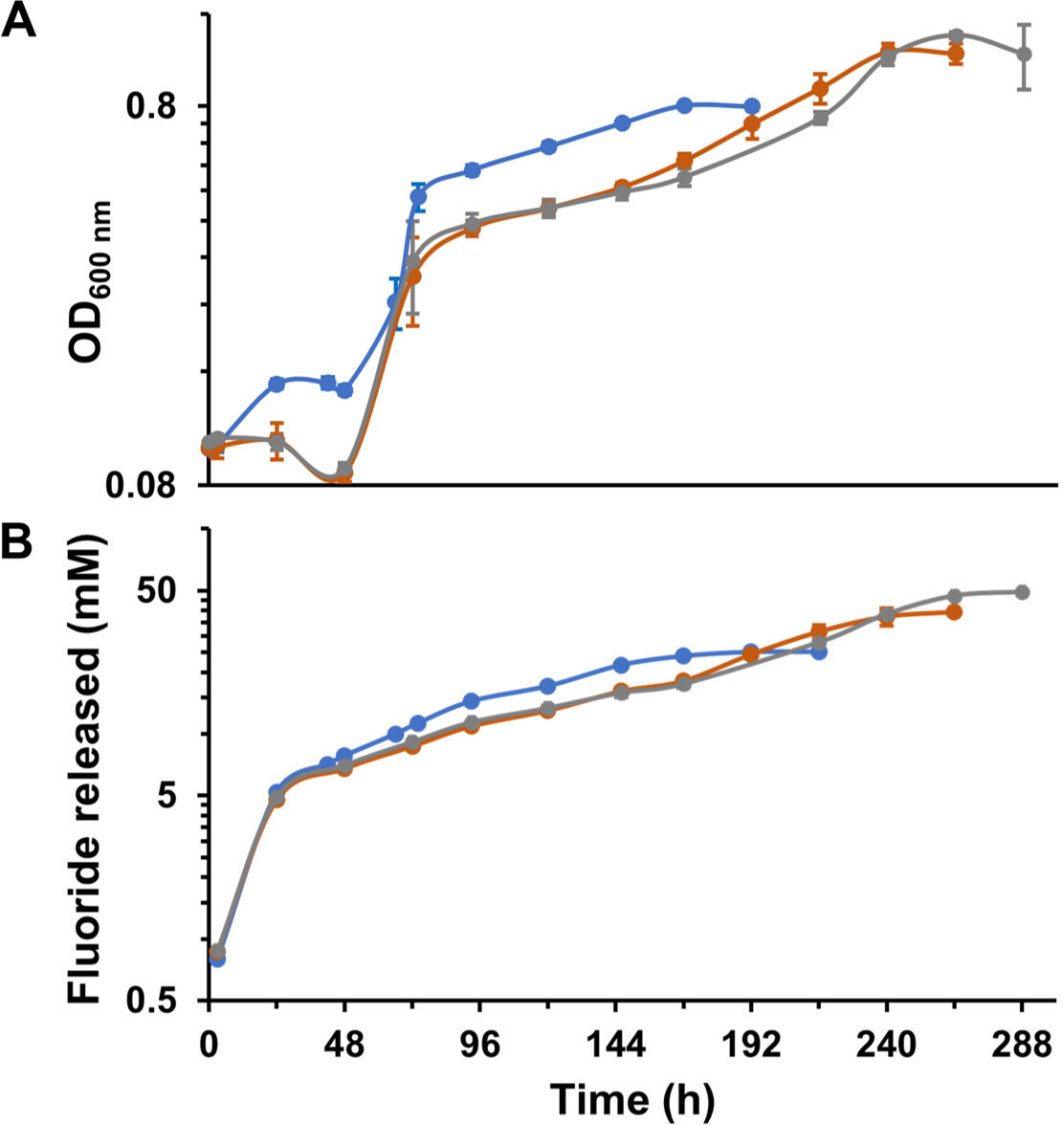
Determining growth stress as determined by the long lag phase and accompanying fluoride release with high concentrations of 2-fluoropropionic acid as the sole carbon source in a minimal medium. (**A**) Growth and (**B**) fluoride release curves of recombinant *P. putida* 12633 with 50 (blue), 80 (orange), or 100 (gray) mM 2-fluoropropionic acid. Error bars represent the standard deviations of three replicates.

When strain 12633 was cultured on 50–100 mM racemic 2-fluoropropionic acid as the sole source of carbon and energy, the culture took 1–2 weeks to grow to completion and showed irregular growth (Fig. 4A). With 50 mM, there was only a minor increase in turbidity over the first 48 h. With 80 and 100 mM 2-fluoropropionic acid, a slight decrease in turbidity was observed over the initial 48 h. This is consistent with the previously observed decrease in colony-forming units seen with 2-fluoropropionic acid. Fluoride was released during this initial non-growth period and attained ~8 mM in all cultures by 48 h. After 48 h, the culture with 50 mM 2-fluoropropionic acid showed the highest OD_600 nm_ (Fig. 4A) and the highest release of fluoride. After 168 h, the 50 mM culture ceased increasing in turbidity and had released the near theoretical limit of fluoride, showing complete consumption of one enantiomer of the substrate. After 168 h, the 80 and 100 mM cultures continued to slowly increase in turbidity and release fluoride. Both attained near complete release of fluoride of 40 and 50 mM, respectively. An attempt to grow the strain on 120 mM of 2-fluoropropionic acid resulted in growth arrest, indicating heightened toxicity to fluoride, the substrate, or a combination of both.

Similar experiments with high levels of α-fluorophenylacetic acid showed that the cells could not process more than 40 mM without observable growth arrest. Given that α-fluorophenylacetic acid is a sevenfold faster substrate for the defluorinase, the more rapid initial intracellular fluoride release would be expected to impose higher stress. Taken together, these data showed that *P. putida* 12633 is capable of degrading very significant (50 mM) levels of a fluorinated substrate but, under those conditions, shows extreme stress leading to atypical and slow growth.

## DISCUSSION

To our knowledge, this is the first report of microbes generating up to 50 mM fluoride via enzymatic defluorination and responding to high levels of fluoride generated directly in the cytoplasm. The defluorination reaction produces fluoride and alcohol, and since HF has a pKa of 3.2 in water, it is expected to be largely ionized at the intracellular pH (39). Some reports of growth on fluorinated compounds, or non-growth biodegradation of PFAS chemicals, have measured fluoride release to be 1–10 mM (19–22). In completely different studies in which fluoride stress was investigated, fluoride salts were added to the growth medium, over a much wider range of 0.1–100 mM (5, 6, 8, 9, 40–43). Under those conditions, the amount of fluoride entering cells is not precisely known, although it is thought that toxic effects can manifest at sub-millimolar concentrations (6, 9, 12, 13, 44).

The *Delftia* enzyme proved to be a useful defluorinase for the present purposes of generating high levels of fluoride intracellularly and making alcohols that support growth as a carbon and energy source. Stable, hydrolytic enzymes catalyzing defluorination are relatively rare, likely due to the paucity of fluorinated natural products (18, 27). Natural products containing more than one fluorine substituent are currently unknown, and highly fluorinated synthetic chemicals are largely resistant to known enzymes. The *Delftia* defluorinase was only reactive with monofluorinated compounds that we tested, but it had sufficiently broad specificity and high activity to generate a relatively high fluoride flux intracellularly with substrates able to support growth. The enzyme could generate both aromatic and aliphatic α-hydroxy acids with an enantioselectivity that allowed product utilization by *Pseudomonas* strain ATCC 12633. The rate with α-fluorophenylacetic acid (14.5 µmol min^−1^ mg^−1^) was as high as many enzymes working on their natural substrates, allowing for a significant flux of fluoride release and significant growth in 24 h. The rate of the enzyme with 2-fluoropropionic acid was nearly an order of magnitude less, allowing a comparison of high flux and lower flux fluoride release on the bacterium.

The observed impacts of fluoride on the cell lag phase, growth yield, and cell morphology are unlikely due to product inhibition of the enzyme by fluoride anion. We tested purified *Delftia* defluorinase in reaction mixtures with fluoride and saw minimal inhibition (Fig. S4). This observation is consistent with homologous defluorinase enzymes that have a structurally analogous active site. In those enzymes, a low affinity for the liberated fluorinated anion was also observed and is proposed to be due to a tryptophan residue that can assist in fluoride transfer out of the active site (15). In our modeling, we see comparable structural features in the *Delftia* defluorinase. Once in a bulk water solvent, fluoride anion is known to be extensively hydrated (39, 45), and significant energy would be required to strip off the water and reenter the enzyme fluorine binding site.

Mitigation of fluoride stress has been shown to be multi-faceted, with some bacteria sensing toxic fluoride with a riboswitch, producing enzymes less sensitive to fluoride, inducing fluoride-specific membrane export proteins, and carrying out other as-yet-to-be defined functions (5, 8, 9). The induction of the membrane export protein-designated CrcB is shown to be highly protective against fluoride (9, 12, 13). CrcB proteins have been purified, crystallized, and tested for activity in liposomes. The latter has demonstrated fluoride export rates as high as 10,000–100,000 s^−1^. Experiments to date have exposed cells to external fluoride by supplementation of the growth medium, and the precise intracellular concentration of fluoride at any one time under those conditions is difficult to determine.

Based on previous studies and genomic comparisons, *P. putida* ATCC 12633 is likely to be representative of many *Pseudomonas* strains with respect to their response to externally added and internally generated fluoride anion. A previous study with *P. putida* KT2440 used a *Tn*-Seq protocol to identify genes involved in mitigating fluoride toxicity (9). The genes identified in that study were found here to also be present in the genome of *P. putida* ATCC 12633 (Table S1). Previous studies had also shown that a knockout of the *crcB* gene, encoding a membrane protein that exports fluoride, makes *P. putida* KT2440 (46) and other bacteria (13) much more sensitive to fluoride. The CrcB protein in strain 12633 is 98% identical to that in strain KT2440, and the gene region is identical (Fig. S5). The CrcB protein is highly conserved in many *Pseudomonas* spp. (Fig. S5; Table S2), suggesting this genus to be generally robust to fluoride at relatively high concentrations. There is still much to be learned about fluoride stress responses. The cell shortening observed here has not previously been reported in response to fluoride but has been observed in *Pseudomonas syringae* under starvation stress conditions (47).

*P. putida* strains have become a focus of biocatalysis and biodegradation, including on the industrial scale (48–50), because of their resistance to various stresses other than fluoride stress. Several studies have focused on individual stress responses. Examples of stressful exposures include solvents (51, 52), desiccation (53), oxidizing agents (54), arsenic (55), and the uncoupling agent 2,4-dinitrophenol (56). One comparison that can be made is the decrease in growth yield from different stresses, which we determined here to be 41% for cytoplasmically released fluoride under the growth conditions used. By comparison, solvent stress in *P. putida* S1 was found to decrease growth yield by 26% (57).

A recent effort sought to define the system-wide transcriptional regulatory network of *P. putida* KT2440 (58), but that study did not include a response to stress from fluoride or organofluorine compound degradation. The present study begins a study of organofluorine compound degradation, which is at a relatively nascent stage, due to the newness of these compounds in the biosphere. Overall, the results suggest that the biodegradation of organofluorine compounds at millimolar concentrations can be achieved with *Pseudomonas* strains, from the perspective of surviving the fluoride stress. There is a parallel interest in using *Pseudomonas* as a vector for the biosynthesis of new fluorinated compounds (9, 46, 59), some of which may serve as substitutes for synthetic fluorinated chemicals. For both biodegradation and biosynthesis purposes, further studies on fluoride stress mitigation are warranted.

## MATERIALS AND METHODS

### Bacterial strains, plasmids, and growth conditions

*Pseudomonas putida* ATCC 12633 was purchased from the American Type Culture Collection (Manassas, VA, USA), and *Escherichia coli* NEB 5-alpha and BL21(DE3) competent cells were purchased from New England Biolabs (Ipswitch, MA, USA). Plasmid pBBR1MCS-2 (60) was purchased from Addgene (Watertown, MA, USA). Strains were grown with shaking at 200 rpm in lysogeny broth (LB) or on various carbon sources in a mineral salts basal (MSB) medium composed of sodium/potassium phosphate buffer (40 mM, pH 6.8), ammonium chloride (1 g/L), and metals and trace elements as described (61), and supplemented with biotin and nicotinic acid at 10 mg/L and thiamine hydrochloride at 5 mg/L. Solid media contained 1.5% agar (LB) or Noble agar (MSB). The media used to grow recombinant strains contained 50 µg/mL kanamycin sulfate (GoldBio, Saint Louis, MO, USA). *Pseudomonas* strains were grown at 28°C or 30°C, and *E. coli* strains were grown at 37°C or as indicated. Cultures were grown in 20 mL aliquots of media in 125 mL baffled flask or as noted. Media were inoculated to a starting OD_600 nm_ of 0.05–0.1 with mid-exponential or stationary phase cells that were washed 1× and concentrated 20–100× with carbon-free MSB. Inocula for growth on α-fluorophenylacetic acid or mandelic acid were grown on 25 mM (*RS*)-mandelic acid in MSB as sole carbon, and inocula for growth on 2-fluoropropionic acid or D-lactic acid were grown on 50 mM D-lactic acid as sole carbon. Growth assays in 96-well plates were conducted in a Tecan (Männedorf, Switzerland) shaking incubator.

### Chemicals

Chemicals were purchased from MilliporeSigma (Saint Louis, MO, USA) or as indicated: (*RS*)-α-fluorophenylacetic acid (Santa Cruz Biotechnology, Dallas, TX, USA), (*RS*)-2-fluoropropionic acid, (*R*)-hydroxypropionic acid (D-lactic acid), 2,2-difluoropropionic acid, α,α-difluoropropionic acid (Oakwood Chemical, Estill, SC, USA), sodium L-lactate, sodium fluorophosphate (Alfa Aesar, Ward Hill, MA, USA), 2-chloro-2-fluoro-acetic acid (AstaTech, Bristol, PA, USA), 2-fluoroacrylic acid (AmBeed, Arlington Heights, IL, USA), and sodium hexafluorophosphate (Chem-Impex, Wood Dale, IL, USA). Benzyl fluoride was synthesized using benzyl alcohol and diethylaminosulfur trifluoride (DAST) via an existing protocol (62) with scale-up and purification as described in the Supplement (Fig. S6).

### Gene cloning and creation of expression strains

Constitutive fluoroacetate dehalogenase expression vectors were created by cloning synthetic gene expression cassettes into plasmid pBRR1MCS-2. Amino acid sequences of fluoroacetate dehalogenases from *Rhodopseudomonas palustris* strain CGA009 (RPA1163) (GenBank accession OPF91568.1) or *Delftia acidovorans* strain B (formerly *Moraxella* sp. strain B) (GenBank accession BAC81979.1) were used to generate codon-optimized gene sequences for expression in *Pseudomonas putida* (Fig. S9) that were synthesized by Integrated DNA Technologies (Coralville, IA, USA). Additional sequences were added to the 5′ end of the coding sequences to include a TEV protease recognition site, codons for a six-histidine tag, a start codon, a ribosomal binding site, the T5 promoter sequence, an AgeI cleavage site, and 27 nt that were complementary to the upstream plasmid arm. Additional sequences were added to the 3′ end of the coding sequences to include an NsiI cleavage site and 32 nt that were complementary to the downstream plasmid arm.

To create the expression vectors, pBRR1MCS-2 was digested with restriction enzymes AgeI and NsiI (New England Biolabs) to remove an 836 bp fragment that contained the MCS and *lac* regulatory elements. Digestion was verified by agarose gel electrophoresis, and the 4.3 kb linearized vector backbone band was excised and purified using a QIAquick Gel Extraction Kit (QIAGEN, Hilden, Germany). Circular expression plasmids were created by combining the purified linearized vector backbone and the synthetic expression cassettes in assembly reactions using an NEB HiFi Assembly kit (New England Biolabs). The reactions were then used to transform high-efficiency NEB 5-alpha competent *E. coli* cells, which were plated onto LB+kanamycin agar plates. Plasmids were isolated from kanamycin-resistant clones using a QIAGEN plasmid miniprep kit, and the presence of the expression cassettes was verified by Sanger sequencing (ACGT, Inc., Wheeling, IL, USA). The plasmids were then used to transform *E. coli* BL21(DE3) cells via the manufacturer’s protocol and *P. putida* ATCC 12633 cells via electroporation (63). For electroporation, cells were grown overnight in LB, harvested in a microcentrifuge at 8,600 × *g*, washed twice with 300 mM sucrose, and then resuspended in 0.05 mL of 300 mM sucrose per mL of culture harvested. Plasmid DNA (0.5 mg) was added to 0.1 mL aliquots of concentrated cells and transferred into 2 mm gap electroporation cuvettes (Fisher Scientific, Pittsburg, PA, USA). Cells were pulsed at 2.5 kV in a Bio-Rad (Hercules, CA, USA) MicroPulser electroporator, recovered with 0.9 mL of SOC medium, and transferred into 14 mL Falcon tubes (Corning, Reynosa, Mexico), which were then incubated for 2 h with shaking (200 rpm) at 28°C. The aliquots of recovered cells were spread onto LB+kanamycin plates and incubated for 48 h. The transformation efficiency was ≥30 CFU/µg plasmid DNA.

### Protein expression and purification

Because the two enzymes expressed differentially in the two host strains, the *Rhodopseudomonas* and *Delftia* enzymes were purified from their *E. coli* BL21(DE3) or *P. putida* ATCC 12633 expression host, respectively. The aliquots (1 L) of LB+kanamycin in 4 L Erlenmeyer flasks were inoculated to a starting OD_600 nm_ of 0.05 with overnight cultures of the expression strains. The cultures were then incubated for 20 h on a shaker at 28°C, and cells were harvested by centrifugation at 4,100 × *g* for 15 min. Cell pellets were resuspended in 15 mL of buffer A (50 mM Tris-Cl+0.2 M NaCl, pH 7.5), a Pierce Protease Inhibitor EDTA-Free Mini tablet (Thermo Scientific, Rockford, IL, USA) was added, the cells were lysed using a French pressure cell (three cycles at 140 MPa), and the crude lysates were centrifuged at 19,000 × *g* for 90 min. The resulting cleared lysates were loaded into a GE Healthcare (Cytiva, Marlborough, MA, USA) AKTA fast protein liquid chromatography (FPLC) system and injected onto a HisTrap HP 5 mL column (Cytiva) that had been charged with Ni^2+^. Unbound proteins were flushed from the column with 98% buffer A+2% buffer B (buffer A+500 mM imidazole, pH 7.5). For *Rhodopseudomonas* enzyme purification, weakly bound proteins were eluted with a wash of 100 mM imidazole (80% buffer A+20% buffer B), and the remaining bound proteins were eluted with a linear gradient from 100 mM to 250 mM imidazole (50% buffer A+50% buffer B) followed by a wash with 250 mM imidazole. Because a significant amount of the his-tagged enzyme was eluted in the 100 mM imidazole wash, the method for *Delftia* enzyme purification was adjusted to a 50 mM imidazole wash (90% buffer A+10% buffer B) to remove weakly bound proteins and then a linear gradient from 50 to 250 mM imidazole to elute the remaining bound proteins. The purity of fractions was assessed by SDS-PAGE, and linear gradient fractions of equivalent purity were pooled. Imidazole was removed by five cycles of concentrating the protein solution to 0.5–1.0 mL with Amicon Ultra (MilliporeSigma) centrifugal filters (10,000 molecular weight cutoff) and then diluting to 15 mL with buffer (20 mM HEPES+0.2 M NaCl, pH 7.5). The protein concentration was measured via the Bradford method with the Bio-Rad (Hercules, CA, USA) Protein Assay Dye Reagent and a standard curve prepared from a commercial bovine serum albumin (BSA) standard (Thermo Scientific). The aliquots of the *Rhodopseudomonas* or *Delftia* enzyme at 13 or 6 µg/mL, respectively, were frozen in liquid nitrogen and then stored at −80°C.

### Enzyme activity assays

The activity of the purified enzymes was measured as net fluoride release from fluorinated substrates using a fluoride ion selective electrode interfaced with an Orion Star A214 pH/ISE meter (Thermo Scientific) calibrated with sodium fluoride standards. Non-natural fluorinated substrates were screened as substrates in 1 mL reactions of 10 mM substrate and 50 µg of purified enzyme in 50 mM Tris-Cl buffer (pH 7.5) incubated at room temp for 1–24 h. The aliquots of the substrate solution without enzyme added were used as controls for spontaneous fluoride release. Reactions to determine specific activity were done at 30°C in 1 mL aliquots of 100 mM Tris-Cl (pH 9.0) with 30 mM substrate and 7–500 µg of purified enzyme and incubated for 10–40 min. Specific activity was calculated from fluoride release in triplicate reactions at three different time points.

### Stereochemistry determinations

To infer which α-fluorophenylacetic enantiomer was reactive with the *Delftia* defluorinase, *P. putida* ATCC 12633 cells constitutively expressing the enzyme were grown on a limiting amount (10 mM) of the racemic substrate in MSB. Aliquots (2 × 100 mL) in 500-mL baffled flasks were inoculated and incubated for 48 h, and then the cells were pelleted by centrifugation as above. The supernatant was filtered with a 0.22 µM PES bottle top filter (Corning, Oneonta, NY, USA), and the pH was adjusted to 2.0 with hydrochloric acid prior to extraction with 3 × 200 mL sequential aliquots of dichloromethane in a separation funnel. The lower (organic) layers were drained from the funnel and pooled, and then, the solvent was removed using a Büchi Rotavapor RE 120 (Flawil, Switzerland) rotary evaporator. The solid material was dried further using a vacuum pump. An aliquot of the dried material was dissolved in deuterochloroform, and a Varian 400-MHz NMR was used to acquire ^1^H and ^19^F spectra, which showed α-fluorophenylacetic acid and water as the only detectable compounds. The remaining dried material was dissolved in 5 mL of deionized water and then analyzed for optical rotation in a Rudolf (Hackettstown, NJ, USA) Autopol III polarimeter at 589 nm.

A real-time spectrophotometric assay, in which the oxidation of D- or L- lactate by commercial D- or L-lactate dehydrogenases (from *Lactobacillus leichmannii* or rabbit muscle, respectively) was coupled to the reduction of NAD^+^ to NADH, was used to identify the product of the *Delftia* defluorinase reaction when combined with racemic 2-fluoropropionic acid. *Delftia* defluorinase (1 mg) was added to 5 mL of 100 mM 2-fluoropropionic acid in 0.1 M Tris-Cl (pH 9.0), and the reaction was incubated overnight at room temperature, after which 50 mM fluoride release was confirmed. To test for the presence of D- or L- lactate, 0.450 mL of the defluorinase reaction was combined with 0.050 mL of 50 mM NAD^+^ in a quartz cuvette, and the formation of NADH was monitored as an increase at 340 nm over time in the absence of lactate dehydrogenase or after addition of 1 unit of D- or L-lactate dehydrogenase. The activity and specificity of both lactate dehydrogenases were verified by testing each separately in similar reactions using 50 mM D- or L-lactate as the substrate with 1 unit of enzyme.

### Active site modeling and substrate docking

The pre-existing AlphaFold (37, 38) structure of the *Delftia* defluorinase (UniProt accession Q01398) was used for modeling interactions between the enzyme and different fluorinated ligands. The AlphaFold structure was aligned to the crystal structure of the *Burkholderia* sp. FA1 FAcD D104A mutant complexed with fluoroacetate (Protein Data Bank ID 3B12) (36), and ligands of interest were fitted to the electron density of the bound fluoroacetate using Coot (64). Restraint dictionaries for ligands were created on the Grade Web Server (65). The protein–ligand complex structures were then subjected to energy minimization and geometric refinement using the Chiron tool (66). Visualization was done in PyMOL (The PyMOL Molecular Graphics System version 2.0, Schrödinger, LLC, New York, NY, USA).

### Growth assays

Growth yields of the *P. putida* ATCC 12633 *Delftia* FAcD expression strain growing on fluorinated or non-fluorinated analogs as sole carbon sources were determined in triplicate with 20 mM of racemic fluorinated substrate (α-fluorophenylacetic acid or 2-fluoropropionic acid), 10 mM of the enzymatic defluorination product [(*R*)-mandelic acid or D-lactic acid], or 10 mM of the defluorination product plus 10 mM sodium fluoride. Stationary phase cells were used as the inocula. Growth was measured as OD_600 nm_ using a Beckman DU-640 spectrophotometer (Beckman-Coulter, Indianapolis, IN, USA), and fluoride release was measured as above. Lag phase determinations were done in a Corning Costar (Kennebunk, ME, USA) 96-well flat-bottom cell culture plate. Aliquots (0.1 mL) of media inoculated with exponential-phase cells were dispensed into wells (10 replicates for each treatment), and then, the plate was shaken at 30°C with OD_600 nm_ readings taken every 20 min. To compare growth on solid media, MSB+Noble agar plates were prepared with 20 mM α-fluorophenylacetic acid or 10 mM (*RS*)-mandelic acid with or without 10 mM sodium fluoride. Noble agar was autoclaved with half of the water and cooled to 50°C and then combined with all remaining components that were prepared as separate solutions, sterilized with 0.22 µM PES membrane syringes (VWR, Radnor, PA, USA) or bottle-top (Corning) filters, and warmed to 50°C. Plates were inoculated by spreading 0.1 mL of mid-exponential phase cells that were grown as above on (*RS*)-mandelic acid and diluted to appropriate density in carbon-free MSB.

### Microscopy

Light micrographs were acquired using a Leica Microsystems (Wetzlar, Germany) DM500 compound microscope and an affixed ICC50 W camera. Images were captured using Leica Application Suite (LAS EZ) software, version 3.4.0. Cell size measurements were done using ImageJ v1.53t (67).

### Identification and similarity of *Pseudomonas* spp. CrcB proteins

Protein sequences encoded in a previously curated set of 251 *Pseudomonas* spp. genomes (68) were downloaded, and potential CrcB proteins were identified using the CrcB-like protein, Camphor Resistance (CrcB) hidden Markov model (InterPro PF02537) with a gathering threshold of 32.9. The significance of the matches was determined using hmmsearch 3.1b2 (69). Pairwise BLAST (70) was used to determine the percent identities of the identified putative CrcB sequences to the CrcB sequence from *P. putida* ATCC 12633 (Table S2).

## SUPPLEMENTAL MATERIAL

Supplemental figures and tables mbio.02785-23-s0001.docx Figures S1 to S7 and Tables S1 and S2.

ASM does not own the copyrights to Supplemental Material that may be linked to, or accessed through, an article. The authors have granted ASM a non-exclusive, world-wide license to publish the Supplemental Material files. Please contact the corresponding author directly for reuse.
