# Supplementary material for: Recombinant Pseudomonas growing on non-natural fluorinated substrates shows stress but overall tolerance to cytoplasmically released fluoride anion
Source: mBio. 2023 Dec 8;15(1):e02785-23. doi: 10.1128/mbio.02785-23 (PMC10790756; doi:10.1128/mbio.02785-23)
Supplement: Supplemental figures and tables — Figures S1 to S7 and Tables S1 and S2. [file mbio.02785-23-s0001.docx]

**SUPPLEMENTAL FIGURES**

**Figure S1**

**
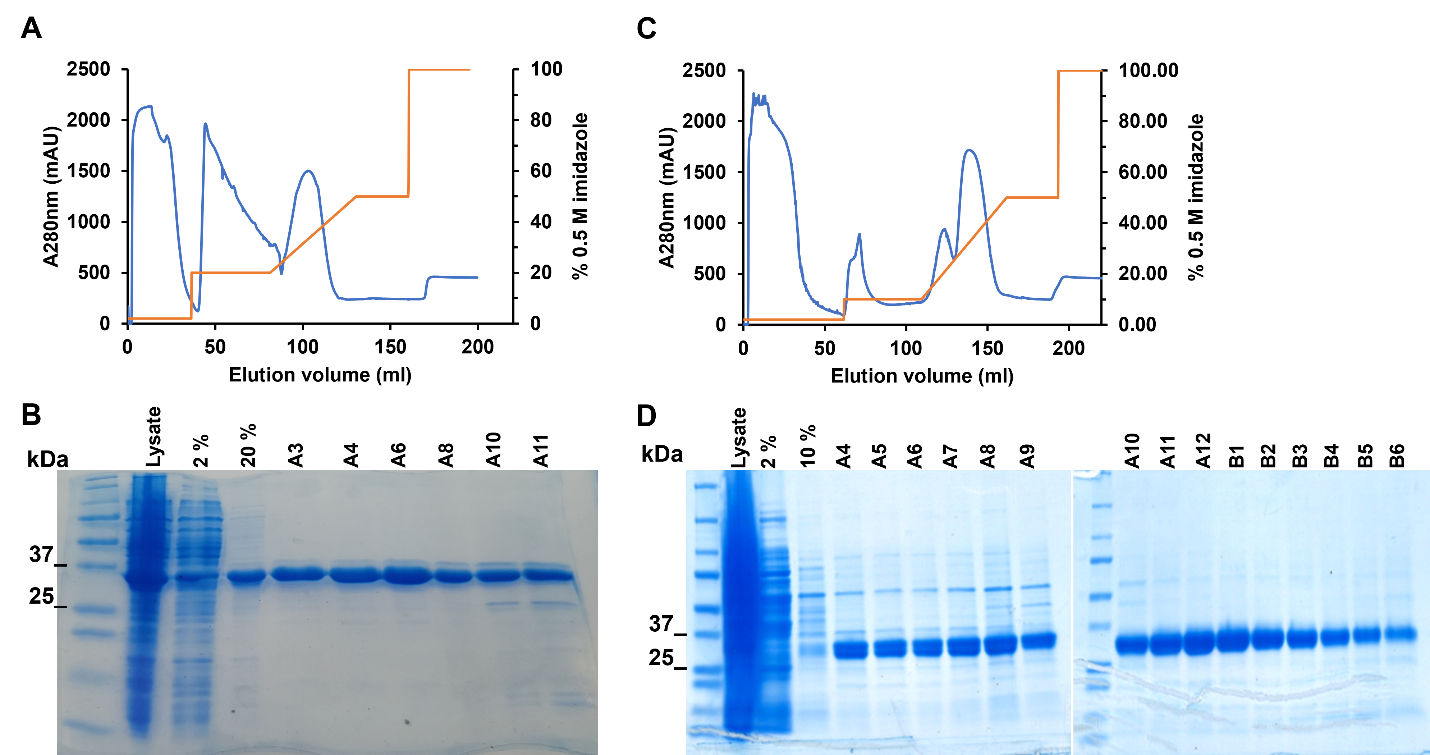
**

**Figure S1**. FPLC nickel-affinity purification chromatograms and SDS-PAGE analyses showing the elution profiles and fraction purity of his-tagged (A,B) *R. palustris* FAcD overexpressed in *E. coli* BL21(DE3) or (C,D) *D. acidovorans* defluorinase overexpressed in *P. putida* ATCC 12633. (A,C) Protein was tracked as absorbance at 280 nm (blue curves) during step washes and linear gradient elution with buffer containing 0.5 M imidazole (orange curves). The apparent double peak during the linear gradient elution in panel D was due to the injection of cleared lysate onto the column in two stages. (B,D) Gel photos show 250 – 10 kDa MW standards, cleared lysate (lysate), step washes at specified percentages of 0.5 M imidazole buffer, and selected 3 ml fractions (letter + numeral, first fraction = A1) collected during the linear gradients. Samples were run on (B) a 12% gel or on (D) two 3 - 15% gradient gels (Bio-Rad). The ranges of fractions pooled for activity assays were (B) A3 – A8 or (D) B1 – B4) for *R. palustris* FAcD or *D. acidovorans* defluorinase, respectively.

**Figure S2**


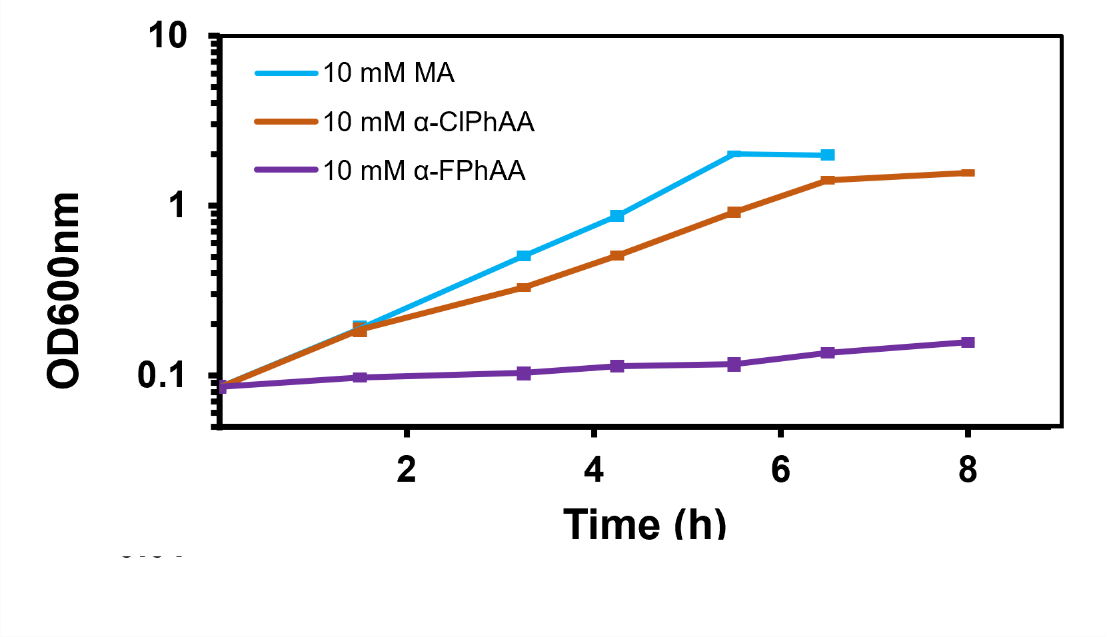


**Figure S2**. Growth of recombinant *P. putida* 12633 expressing the *Delftia* defluorinase on mandelic acid (MA), α-chlorophenylacetic acid (α -ClPhAA), or α-fluorophenylacetic acid (α-FPhAA) as the sole carbon and energy source in minimal medium at the concentrations indicated. Error bars represent the standard deviations of three biological replicates. Data collection stopped when cultures growing on 2-ClPhAA reached stationary phase. The experiment was repeated once and similar results were obtained.

**Figure S3**


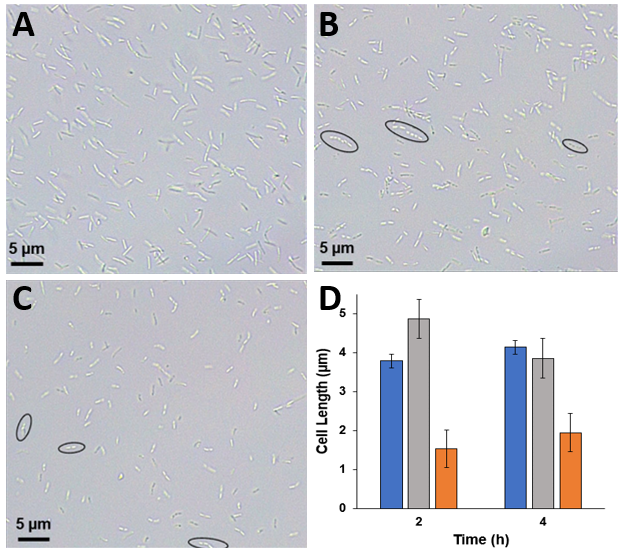


**FIG S3** (A-C) Light micrographs (400× magnification) showing cell morphologies of the *P. putida* 12633 *Delftia* defluorinase expression strain during growth in liquid minimal media with (A,B) 12.5 mM (*RS*)-mandelic acid or (C) (*RS*)-fluorophenylacetic acid as the sole carbon and energy source and (B) with 12.5 mM NaF added as a source of extracellular fluoride. Micrographs were taken during exponential growth at the following times/cell densities: (A) 5 h/ OD_600nm_ = 1.1, (B) 7 h/ OD_600nm_ = 1.1, (C) 25 h/ OD_600nm_ = 0.67. Circled areas show morphological types correlated with the presence of fluoride ions that were (B) added to the medium before inoculation or (C) released from the fluorinated substrate during growth. (D) Cell lengths of *P. putida* 12633 wild-type cells growing on 20 mM (*RS*)-mandelic acid in liquid minimal medium without chloride or fluoride supplementation (blue columns) or with 50 mM NaCl (gray columns) or NaF (orange columns) added to the medium. Cell lengths were determined from light micrographs at 400× magnification using ImageJ. Error bars represent the standard error of measurements from 10 cells.

**Figure S4**


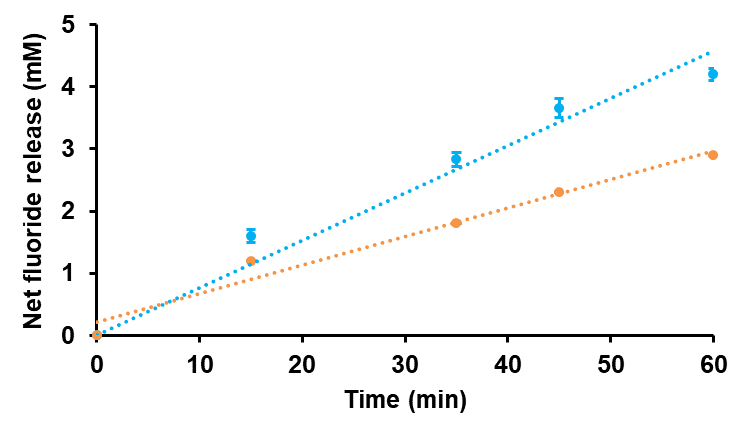


**Figure S4**. Activity (measured as net fluoride released during incubation) of purified *Delftia* defluorinase on 20 mM α-fluorophenylacetic acid in the presence (orange) or absence (blue) of 5 mM fluoride anion (from 5 mM NaF). Error bars show the standard deviations of 3 replicate reactions at each time point. The experiment was repeated once and it produced similar results.

.

**Figure S5_­_**


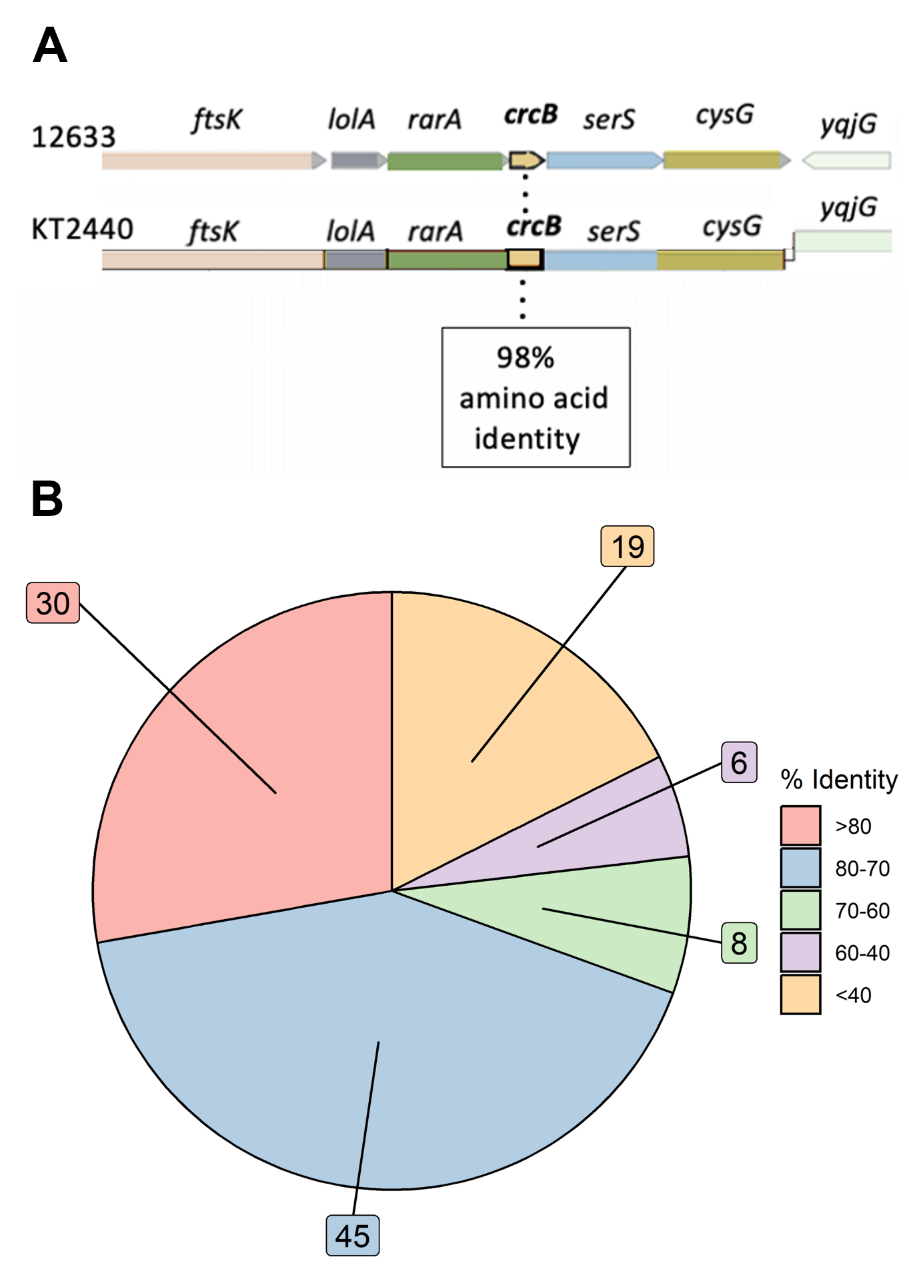


**Figure S5** Basis of fluoride resistance in *P. putida strain* 12633 and presence of the fluoride exporter CrcB in 108 *Pseudomonas* spp. strains. (A) Fluoride resistance operon in *P. putida* strain 12633 (this study) vs. KT2440 (characterized). (B) Potential CrcB proteins were identified as described in the Methods, compared to the CrcB protein from strain 12633, and the percent identity distribution was plotted as a pie chart. The number of proteins within each percent identity range are indicated in the boxes outside of the pie chart. Source strains, protein accession numbers, and similarity scores are in Table S2.

**Figure S6**

CH_2_Cl_2_/-5° pH 12 aq. KOH

Et_2_NSF_3_ + C_6_H_5_CH_2_OH ───────→ C_6_H_5_CH_2_F + HF + Et_2_NS(O)F ( ─────────→ Et_2_NSO_2_K)

“DAST” “DATF”

(only slowly reacts with neutral H_2_O)

**Figure S6.** Scale-up (35×) of the published benzyl fluoride synthesis protocol (Middleton) via the reaction of benzyl alcohol with diethylaminosulfur trifluoride (DAST). A solution of 31 g (0.29 mole) of benzyl alcohol in 50 mL of CH_2_Cl_2_ was slowly added over 1 hr to a stirred cold (-5°C) solution of 52 g (8% excess) of DAST in 100 mL of anhydrous CH_2_Cl_2_ . No exotherm was noted on warming to RT. To remove excess DAST and the co-product diethylamino thionyl fluoride (DATF), the reaction mix was poured onto 500 g of ice then made strongly basic (pH 12, to destroy DATF) by adding 20% aq. KOH and stirring vigorously for 5 minutes. The organic phase was washed twice with 500 mL H_2_O, the second wash was made weakly acidic (HCl). *(Before proceeding to subsequent distillations, a dried (MgSO_4_) sample of the organic phase must be checked by ^19^F NMR for absence of DATF. This spectrum (1:2 in CDCl_3_), must show no remaining singlet near +60 ppm for DATF (which slowly hydrolyzes releasing HF and SO_2_) and should show a strong triplet (-205 ppm) for benzyl fluoride surrounded by weak triplets for higher boiling co-products: o-, m-, and p-isomers of N,N-diethylaminosulfinyl benzyl fluoride, (DASBF) Et_2_NS(O)C_6_H_4_CH_2_F.)* The combined product solutions and washes free of DATF (about 250 mL) were dried (filtered through 25 g MgSO_4_) then carefully distilled through a 6-inch vacuum jacketed column to remove most of the solvent. The crude distillation pot residue (~30 mL) was cooled and vacuum distilled (10-15 mm Hg) to chilled (-20 to -40°C) receiver collecting 32 g of distillate, which was about 53 wt % benzyl fluoride in CH_2_Cl_2_, free of higher boiling DASBF co-products. Normal column distillation at atmospheric pressure reduced the residue to 18.5 g of 96 wt % pure benzyl fluoride. Slow nitrogen flow evaporation over 1.5 hours left 16.5 g (50% yield) of 97 wt % pure benzyl fluoride (3 wt % CH_2_Cl_2_).

**References**

Middleton WJ. 1975. New Fluorinating Agents. Dialkylaminosulfur Fluorides *J Or. Chem* 40:574-578.

**Figure S7**

>*Rhodopseudomonas palustris* ATCC BAA-98 FAcD

ATGCCCGACCTGGCAGACTTGTTTCCAGGCTTTGGCAGCGAATGGATTAACACTTCCAGC

GGTCGTATCTTCGCACGTGTCGGTGGTGATGGTCCACCGTTGTTGCTGCTCCACGGTTTC

CCACAAACCCATGTGATGTGGCACCGCGTGGCCCCAAAATTGGCAGAGCGCTTCAAGGTG

ATTGTCGCGGATCTCCCGGGCTATGGCTGGTCCGATATGCCAGAATCCGATGAGCAACAT

ACCCCCTATACCAAGCGCGCTATGGCTAAGCAACTCATTGAAGCAATGGAACAATTGGGT

CATGTTCATTTTGCATTGGCTGGTCATGATCGTGGGGCACGCGTCAGCTACCGCTTGGCA

CTGGATTCCCCGGGCCGCCTCTCCAAATTGGCAGTGCTGGACATTTTGCCCACGTATGAA

TACTGGCAGCGCATGAACCGCGCATACGCGCTGAAGATTTATCACTGGAGCTTTTTGGCG

CAACCGGCCCCACTGCCGGAAAACCTGTTGGGGGGTGACCCTGACTTCTACGTCAAGGCG

AAACTGGCATCCTGGACCCGTGCGGGTGATTTGTCGGCCTTCGATCCGCGGGCTGTGGAG

CACTACCGTATTGCGTTTGCAGATCCGATGCGCCGCCACGTGATGTGTGAAGACTACCGG

GCAGGTGCTTATGCCGACTTCGAACACGACAAGATTGATGTGGAGGCTGGGAATAAGATT

CCAGTTCCAATGTTGGCACTGTGGGGTGCATCGGGTATTGCACAATCGGCAGCAACGCCG

TTGGATGTATGGCGTAAATGGGCAAGCGATGTCCAAGGTGCGCCGATCGAGAGCGGCCAT

TTCTTGCCTGAAGAAGCCCCTGATCAGACGGCGGAGGCGCTGGTGCGGTTTTTCAGCGCT

GCACCTTGA

>*Delftia acidovorans* strain B defluorinase

ATGGACTTTCCGGGGTTCAAAAACTCCACGGTGACGGTCGATGGGGTCGACATTGCTTAC

ACCGTCTCCGGGGAAGGCCCGCCAGTCTTGATGTTGCATGGGTTCCCACAAAATCGCGCC

ATGTGGGCTCGTGTGGCACCGCAACTGGCTGAGCACCACACGGTCGTCTGTGCTGACTTG

CGCGGCTATGGTGACTCCGACAAGCCCAAGTGTTTGCCAGATCGCTCCAATTACTCGTTC

CGCACCTTTGCTCACGACCAACTCTGTGTGATGCGGCACTTGGGGTTTGAGCGTTTCCAT

CTGGTGGGCCATGACCGGGGGGGCCGTACGGGCCATCGTATGGCATTGGATCATCCTGAA

GCCGTGCTGAGCTTGACGGTGATGGATATCGTGCCCACGTATGCTATGTTCATGAATACG

AACCGTTTGGTGGCGGCGTCGTACTGGCACTGGTATTTTTTGCAGCAACCTGAGCCGTTC

CCAGAACACATGATTGGGCAAGATCCGGACTTCTTTTACGAGACGTGTCTCTTCGGGTGG

GGCGCAACGAAAGTGTCCGATTTCGACCAGCAGATGCTCAACGCCTACCGTGAATCGTGG

CGTAATCCCGCAATGATTCACGGTTCCTGTAGCGATTACCGCGCCGCTGCCACGATTGAT

CTGGAGCACGACTCGGCCGATATTCAACGCAAGGTGGAATGCCCAACGCTGGTGTTTTAT

GGTAGCAAAGGTCAAATGGGGCAACTGTTCGACATTCCAGCTGAGTGGGCGAAGCGCTGT

AATAACACGACCAACGCCAGCCTCCCCGGTGGGCATTTCTTCGTGGACCAATTTCCCGCC

GAGACCTCCGAAATTTTGTTGAAATTTTTGGCGCGGAACGGGTGA

**Figure S7.** Codon-optimized (for *Pseudomonas putida*) gene sequences used to overexpress the encoded fluoroacetate dehalogenases.

**SUPPLEMENTAL TABLES**

**Table S1.** Annotated genes in *Pseudomonas putida* ATCC 12633 relevant to this study.

| **Annotation** | **Gene** | **Genome locus tag** | **DNA sequence start** | **DNA sequence end** | **Translated sequence accession** |
| --- | --- | --- | --- | --- | --- |
| **Mandelate or phenylacetic acid metabolism** |  |  |  |  |  |
| mandelate racemase | mdlA | NP430_24595 | 5472034 | 5473113 | UUI34657.1 |
| alpha-hydroxy-acid oxidizing protein | mdlB | NP430_24590 | 5470856 | 5472037 | UUI34656.1 |
| benzoylformate decarboxylase | mdlC | NP430_24585 | 5469261 | 5470847 | UUI34655.1 |
| aldehyde dehydrogenase family protein | mdlD | NP430_24570 | 5465403 | 5466713 | UUI34652.1 |
| benzoate 1,2-dioxygenase large subunit | benA/cbdA | NP430_14285 | 3188156 | 3189514 | UUI32718.1 |
| benzoate 1,2-dioxygenase small subunit | benB/cbdB | NP430_14280 | 3187674 | 3188159 | UUI32717.1 |
| benzoate 1,2-dioxygenase electron transfer component | benC | NP430_14275 | 3186591 | 3187601 | UUI32716.1 |
| 1,6-dihydroxycyclohexa-2,4-diene-1-carboxylate dehydrogenase | benD | NP430_14270 | 3185636 | 3186397 | UUI32715.1 |
| catechol 1,2-dioxygenase | catA | NP430_16390 | 3674207 | 3675142 | UUI33121.1 |
| muconate cycloisomerase family protein | catB | NP430_16400 | 3675498 | 3676619 | UUI33123.1 |
| muconolactone Delta-isomerase | catC | NP430_16395 | 3675186 | 3675476 | UUI33122.1 |
| alpha/beta hydrolase | catD | NP430_20665 | 4581563 | 4582375 | UUI33924.1 |
| 3-oxoadipate enol-lactonase | pcaD | NP430_22490 | 5014586 | 5015377 | UUI34265.1 |
| 3-oxoadipyl-CoA thiolase | pcaF | NP430_22505 | 5018226 | 5019428 | UUI34268.1 |
| 3-oxoacid CoA-transferase subunit A | pcaI | NP430_18175 | 4032886 | 4033581 | UUI33457.1 |
| 3-oxoacid CoA-transferase subunit B | pcaJ | NP430_18180 | 4033590 | 4034231 | UUI33458.1 |
| cation acetate symporter | actP | NP430_13985 | 3125026 | 3126588 | UUI32663.1 |
| **Lactate metabolism** |  |  |  |  |  |
| DNA-binding transcriptional dual regulator | lldR | NP430_24130 | 5367857 | 5368624 | UUI34575.1 |
| Lactate permease LctP family transporter | lldP | NP430_24135 | 5368879 | 5370549 | UUI34576.1 |
| FMN-dependent L-lactate dehydrogenase | lldD | NP430_24140 | 5370613 | 5371758 | UUI34577.1 |
| FAD-binding oxidoreductase (D-lactate dehydrogenase) | lldE | NP430_24145 | 5371834 | 5374644 | UUI34578.1 |
| DNA-binding transcriptional regulator | glcC | NP430_16760 | 3763780 | 3764544 | UUI33194.1 |
| glycolate oxidase subunit FAD binding domain*^a^* | glcD | NP430_16765 | 3764740 | 3766239 | UUI33195.1 |
| glycolate oxidase subunit FAD binding domain*^a^* | glcE | NP430_16770 | 3766239 | 3767291 | UUI33196.1 |
| glycolate oxidase subunit 4Fe-4S dicluster domain*^a^* | glcF | NP430_16775 | 3767301 | 3768536 | UUI33197.1 |
| NAD-dependent D-lactate dehydrogenase*^b^* | ldhA | NP430_06220 | 1392639 | 1393628 | UUI36441.1 |
| pyruvate kinase | pyk | NP430_19710 | 4384502 | 4385917 | UUI33744.1 |
| pyruvate dehydrogenase (acetyl-transferring), homodimeric type | aceE | NP430_01915 | 452554 | 455199 | UUI35631.1 |
| dihydrolipoyllysine-residue acetyltransferase | aceF/acoC | NP430_01910 | 450903 | 452540 | UUI35630.1 |
| **Fluoride tolerance associated (Calero *et al.)*** |  |  |  |  |  |
| fluoride efflux transporter CrcB | crcB | NP430_18260 | 4045344 | 4045718 | UUI33471.1 |
| replication-associated recombination protein A | rarA | NP430_18265 | 4045715 | 4047040 | UUI33472.1 |
| anti-sigma factor | prtR | NP430_12320 | 2750715 | 2751467 | UUI37583.1 |
| retention module-containing protein |  | NP430_00865 | 186921 | 205874 | UUI35430.1 |
| type I secretion C-terminal target domain-containing protein |  | NP430_00870 | 205943 | 222631 | UUI35431.1 |
| EAL domain-containing protein | lapD | NP430_00850 | 180301 | 182247 | UUI35428.1 |
| transcriptional regulator FleQ | fleQ | NP430_20090 | 4467002 | 4468477 | UUI33818.1 |

*^a^*Subunits that comprise a complex that functions as a D-lactate dehydrogenase. (Zhang *et al*.)

*^b^*Not required for D-lactate assimilation. Catalyzes the reverse reaction (NADH-dependent pyruvate reduction to D-lactate) at physiological pH. NAD-dependent lactate oxidation to pyruvate occurs with high lactate concentrations at high pH. (Zhang *et al*.)

**References**

Calero P, Gurdo N, Nikel PI. 2022. Role of the CrcB transporter of *Pseudomonas putida* in the multi-level stress response elicited by mineral fluoride. *Environ Microbiol* 24:5082-5104. doi: 10.1111/1462-2920.16110

Zhang Y, Jiang T, Sheng B, Long Y, Gao C, Ma C, Xu P. 2016. Coexistence of two D-lactate-utilizing systems in *Pseudomonas putida* KT2440. *Environ Microbiol Rep* 5:699-707. doi: 10.1111/1758-2229.12429.

**Table S2.** CrcB homologs identified in a set of 251 *Pseudomonas* genomes, sorted by percent identity (BLAST) (highest to lowest) of amino acid sequences to the strain 12633 CrcB sequence. Three sequences from *Stutzerimonas* strains (recently removed from genus *Pseudomonas*) (Lalucat *et al.*) were also included.

| **Strain name** | **taxa_id** | **genome_id** | **prot_id** | **hmm_eval** | **hmm_score** | **BLAST % identity** | **length** | **blast_eval** |
| --- | --- | --- | --- | --- | --- | --- | --- | --- |
| *Pseudomonas putida* NBRC 14164 DNA, complete genome | 1211579 | AP013070.1 | BAN53605.1 | 1.40E-23 | 87.5 | 100 | 124 | 2.44E-82 |
| *Pseudomonas kurunegalensis* strain RW1P2 1, whole genome shotgun sequence | 485880 | NZ_JABWSB020000001.1 | HU758_RS00595 | 1.80E-23 | 87.1 | 98.387 | 124 | 1.05E-80 |
| *Pseudomonas mosselii* DSM 17497 Q380DRAFT_scaffold00001.1, whole genome shotgun sequence | 1403339 | NZ_KK211238.1 | WP_028692383.1 | 7.80E-24 | 88.3 | 98.387 | 124 | 1.82E-80 |
| *Pseudomonas entomophila* L48, complete sequence | 384676 | NC_008027.1 | WP_011533436.1 | 8.20E-24 | 88.2 | 97.581 | 124 | 2.17E-80 |
| *Pseudomonas maumuensis* strain COW77 chromosome, complete genome | 2842354 | NZ_CP077077.1 | WP_023630958.1 | 8.90E-24 | 88.1 | 96.774 | 124 | 2.65E-79 |
| *Pseudomonas promysalinigenes* strain RW10S1 chromosome, complete genome | 485898 | NZ_CP077094.1 | WP_060477776.1 | 1.10E-23 | 87.8 | 95.968 | 124 | 9.79E-79 |
| *Pseudomonas muyukensis* strain COW39 chromosome, complete genome | 2842357 | NZ_CP077073.1 | WP_217847464.1 | 5.70E-24 | 88.7 | 95.161 | 124 | 5.91E-79 |
| *Pseudomonas asiatica* strain RYU5 RYU5_unitig_0, whole genome shotgun sequence | 2219225 | NZ_BLJF01000001.1 | WP_015270966.1 | 1.70E-23 | 87.2 | 93.548 | 124 | 3.91E-77 |
| *Pseudomonas kermanshahensis* strain SWRI100 1, whole genome shotgun sequence | 2745482 | NZ_JABWRY020000001.1 | WP_085273002.1 | 1.40E-23 | 87.5 | 92.742 | 124 | 8.82E-69 |
| *Pseudomonas xantholysinigenes* strain RW9S1A chromosome, complete genome | 2745490 | NZ_CP077095.1 | WP_186661935.1 | 1.70E-23 | 87.2 | 91.935 | 124 | 9.22E-76 |
| P*seudomonas anuradhapurensis* strain RD8MR3 chromosome, complete genome | 485870 | NZ_CP077097.1 | WP_186674646.1 | 1.70E-23 | 87.2 | 91.935 | 124 | 1.72E-76 |
| *Pseudomonas xanthosomatis* strain COR54 chromosome, complete genome | 2842356 | NZ_CP077075.1 | WP_217884540.1 | 6.20E-24 | 88.6 | 91.129 | 124 | 7.18E-67 |
| *Pseudomonas oryzicola* strain RD9SR1 1, whole genome shotgun sequence | 485876 | NZ_JABWRZ020000001.1 | WP_189665432.1 | 1.50E-23 | 87.3 | 90.909 | 121 | 3.34E-74 |
| *Pseudomonas fakonensis* strain COW40 chromosome, complete genome | 2842355 | NZ_CP077076.1 | WP_217842985.1 | 4.80E-24 | 89 | 90.323 | 124 | 6.65E-67 |
| *Pseudomonas alkylphenolica* strain KL28 chromosome, complete genome | 237609 | NZ_CP009048.1 | WP_038612780.1 | 1.00E-23 | 87.9 | 88.71 | 124 | 3.06E-65 |
| *Pseudomonas urmiensis* strain SWRI10 1, whole genome shotgun sequence | 2745493 | NZ_JABWRE020000001.1 | WP_186557188.1 | 1.30E-23 | 87.6 | 88.71 | 124 | 2.45E-66 |
| *Pseudomonas tructae* strain SNU WT1 chromosome, complete genome | 2518644 | NZ_CP035952.1 | WP_130264089.1 | 1.30E-23 | 87.5 | 87.903 | 124 | 1.89E-64 |
| *Pseudomonas shirazensis* strain SWRI56 1, whole genome shotgun sequence | 2745494 | NZ_JABWRD020000001.1 | WP_186698894.1 | 1.30E-23 | 87.5 | 87.903 | 124 | 8.75E-75 |
| *Pseudomonas koreensis* strain LMG 21318 chromosome I | 198620 | NZ_LT629687.1 | WP_083366296.1 | 3.70E-23 | 86.1 | 82.114 | 123 | 2.56E-67 |
| *Pseudomonas ogarae*, complete sequence | 1114970 | NC_016830.1 | WP_014337695.1 | 8.10E-24 | 88.2 | 81.301 | 123 | 1.71E-66 |
| *Pseudomonas versuta* strain L10.10 chromosome, complete genome | 1788301 | NZ_CP012676.1 | WP_060691377.1 | 1.20E-23 | 87.7 | 81.301 | 123 | 8.37E-67 |
| *Pseudomonas viciae* strain 11K1 chromosome, complete genome | 2505979 | NZ_CP035088.1 | WP_014337695.1 | 8.10E-24 | 88.2 | 81.301 | 123 | 1.71E-66 |
| *Pseudomonas zarinae* strain SWRI108 chromosome, complete genome | 2745498 | NZ_CP077086.1 | WP_095962337.1 | 9.40E-24 | 88 | 81.301 | 123 | 8.19E-67 |
| *Pseudomonas glycinae* strain MS586 chromosome, complete genome | 1785145 | NZ_CP014205.2 | WP_064380678.1 | 3.50E-24 | 89.4 | 80.488 | 123 | 4.68E-66 |
| *Pseudomonas gozinkensis* strain IzPS32d chromosome, complete genome | 2774461 | NZ_CP062253.1 | WP_007958188.1 | 4.00E-24 | 89.2 | 80.488 | 123 | 2.92E-66 |
| *Pseudomonas monsensis* strain PGSB 8459 chromosome, complete genome | 2745509 | NZ_CP077087.1 | WP_123462490.1 | 8.30E-24 | 88.2 | 80.488 | 123 | 4.11E-66 |
| *Pseudomonas bijieensis* strain SP1 chromosome, complete genome | 2681983 | NZ_CP097108.1 | WP_013693703.1 | 4.50E-23 | 85.8 | 80.488 | 123 | 1.14E-66 |
| *Pseudomonas marvdashtae* strain SWRI102 1, whole genome shotgun sequence | 2745500 | NZ_JABWQX020000001.1 | WP_018608522.1 | 8.60E-24 | 88.1 | 80.488 | 123 | 2.37E-66 |
| *Pseudomonas zanjanensis* strain SWRI12 1, whole genome shotgun sequence | 2745496 | NZ_JABWRB020000001.1 | WP_018608522.1 | 8.60E-24 | 88.1 | 80.488 | 123 | 2.37E-66 |
| *Pseudomonas arsenicoxydans* strain CECT 7543 chromosome I | 702115 | NZ_LT629705.1 | WP_090185602.1 | 1.10E-23 | 87.8 | 80.488 | 123 | 1.89E-65 |
| *Pseudomonas tohonis* strain TUM18999 chromosome, complete genome | 2725477 | NZ_AP023189.1 | WP_111262154.1 | 3.20E-24 | 89.5 | 79.839 | 124 | 3.88E-67 |
| *Pseudomonas protegens* CHA0, complete genome | 1124983 | CP003190.1 | AGL85701.1 | 2.80E-23 | 86.5 | 79.675 | 123 | 5.12E-65 |
| *Pseudomonas brassicacearum* strain BS3663 genome assembly, chromosome: I | 930166 | LT629713.1 | SDP66397.1 | 4.70E-23 | 85.8 | 79.675 | 123 | 4.15E-66 |
| *Pseudomonas mediterranea* strain DSM 16733 genome assembly, chromosome: I | 183795 | LT629790.1 | SDU50979.1 | 7.90E-23 | 85.1 | 79.675 | 123 | 4.02E-64 |
| *Pseudomonas silesiensis* strain A3 chromosome, complete genome | 1853130 | NZ_CP014870.1 | WP_064678726.1 | 1.30E-23 | 87.5 | 79.675 | 123 | 3.30E-65 |
| *Pseudomonas germanica* strain FIT28 chromosome, complete genome | 2815720 | NZ_CP071586.1 | WP_095048067.1 | 1.10E-23 | 87.8 | 79.675 | 123 | 2.20E-65 |
| *Pseudomonas salmasensis* strain SWRI126 chromosome, complete genome | 2745514 | NZ_CP077083.1 | WP_003192080.1 | 6.70E-24 | 88.5 | 79.675 | 123 | 4.02E-66 |
| *Pseudomonas zeae* strain OE 48.2 chromosome, complete genome | 2745510 | NZ_CP077090.1 | WP_007908079.1 | 1.10E-23 | 87.8 | 79.675 | 123 | 1.08E-65 |
| *Pseudomonas reinekei* strain DSM 18361 chromosome I | 395598 | NZ_LT629709.1 | WP_075947929.1 | 7.40E-24 | 88.4 | 79.675 | 123 | 6.95E-66 |
| *Pseudomonas umsongensis* strain LMG 21317 chromosome I | 198618 | NZ_LT629767.1 | WP_020799521.1 | 3.10E-23 | 86.3 | 79.675 | 123 | 3.93E-66 |
| *Pseudomonas synxantha* strain LMG 2190 genome assembly, chromosome: I | 47883 | LT629786.1 | SDU17829.1 | 1.00E-23 | 87.9 | 78.862 | 123 | 7.43E-66 |
| *Pseudomonas mucidolens* strain LMG 2223 genome assembly, chromosome: I | 46679 | LT629802.1 | SDU91314.1 | 1.20E-23 | 87.7 | 78.862 | 123 | 1.72E-51 |
| *Pseudomonas paracarnis* strain UBT403, whole genome shotgun sequence | 2750625 | NZ_CAJFCM010000001.1 | WP_014718898.1 | 4.00E-24 | 89.2 | 78.862 | 123 | 1.48E-65 |
| *Pseudomonas corrugata* strain RM1-1-4 chromosome, complete genome | 47879 | NZ_CP014262.1 | WP_024779294.1 | 2.10E-23 | 86.9 | 78.862 | 123 | 9.05E-64 |
| *Pseudomonas asgharzadehiana* strain SWRI132 chromosome, complete genome | 2842349 | NZ_CP077079.1 | WP_017527197.1 | 2.40E-23 | 86.7 | 78.862 | 123 | 2.59E-66 |
| *Pseudomonas alvandae* strain SWRI17 chromosome, complete genome | 2842348 | NZ_CP077080.1 | WP_030141344.1 | 1.30E-23 | 87.5 | 78.862 | 123 | 3.27E-64 |
| *Pseudomonas deceptionensis* strain LMG 25555, whole genome shotgun sequence | 882211 | NZ_FNUD01000002.1 | WP_048358554.1 | 1.00E-23 | 87.9 | 78.862 | 123 | 8.56E-66 |
| *Pseudomonas laurylsulfatiphila* strain AP3_16 contig1, whole genome shotgun sequence | 2011015 | NZ_NIRS01000001.1 | WP_102673993.1 | 7.90E-24 | 88.3 | 78.862 | 123 | 1.89E-65 |
| *Pseudomonas baetica* strain LMG 25716 Ga0070643_11, whole genome shotgun sequence | 674054 | NZ_PHHE01000001.1 | WP_095189687.1 | 8.20E-24 | 88.2 | 78.862 | 123 | 4.30E-64 |
| *Pseudomonas triticicola* strain SWRI88 1, whole genome shotgun sequence | 2842345 | NZ_JAHSTX010000001.1 | WP_166554690.1 | 1.60E-23 | 87.3 | 78.333 | 120 | 1.12E-62 |
| *Pseudomonas granadensis* strain LMG 27940 genome assembly, chromosome: I | 1421430 | LT629778.1 | SDT26486.1 | 3.30E-23 | 86.3 | 78.049 | 123 | 2.68E-65 |
| *Pseudomonas azerbaijanorientalis* strain SWRI123 chromosome, complete genome | 2842350 | NZ_CP077078.1 | WP_008049383.1 | 4.80E-24 | 89 | 78.049 | 123 | 7.76E-65 |
| *Pseudomonas shahriarae* strain SWRI52 chromosome, complete genome | 2745512 | NZ_CP077085.1 | WP_057441422.1 | 6.10E-24 | 88.6 | 78.049 | 123 | 7.77E-59 |
| *Pseudomonas tensinigenes* strain ZA 5.3 chromosome, complete genome | 2745511 | NZ_CP077089.1 | WP_095118232.1 | 5.80E-24 | 88.7 | 78.049 | 123 | 1.26E-63 |
| *Pseudomonas hamedanensis* strain SWRI65 chromosome, complete genome | 2745504 | NZ_CP077091.1 | WP_186551821.1 | 2.10E-23 | 86.9 | 78.049 | 123 | 6.31E-64 |
| *Pseudomonas iranensis* strain SWRI54 chromosome, complete genome | 2745503 | NZ_CP077092.1 | WP_016775261.1 | 1.70E-23 | 87.2 | 78.049 | 123 | 6.96E-64 |
| *Pseudomonas lalucatii* strain R1b54 1, whole genome shotgun sequence | 1424203 | NZ_JADPMV010000001.1 | WP_213637891.1 | 3.70E-24 | 89.3 | 77.57 | 107 | 6.09E-55 |
| *Pseudomonas furukawaii* strain KF707 chromosome, complete genome | 1149133 | NZ_AP014862.1 | WP_004421855.1 | 2.10E-22 | 83.7 | 77.236 | 123 | 9.37E-62 |
| *Pseudomonas frederiksbergensis* strain AS1 chromosome, complete genome | 104087 | NZ_CP018319.1 | WP_074878168.1 | 2.70E-24 | 89.8 | 77.236 | 123 | 7.19E-64 |
| *Pseudomonas taetrolens* strain NCTC10697 chromosome 1, complete sequence | 47884 | NZ_LS483370.1 | WP_048382509.1 | 2.20E-23 | 86.8 | 77.236 | 123 | 1.99E-65 |
| *Pseudomonas caricapapayae* strain CCUG 32775T contig_0000001, whole genome shotgun sequence | 46678 | NZ_VXJY01000001.1 | WP_002553993.1 | 5.40E-22 | 82.4 | 77.236 | 123 | 1.26E-62 |
| *Pseudomonas lalkuanensis* strain PE08 chromosome, complete genome | 2604832 | NZ_CP043311.1 | WP_151134051.1 | 1.60E-22 | 84 | 76.423 | 123 | 8.58E-63 |
| *Pseudomonas guangdongensis* strain CCTCC 2012022 genome assembly, chromosome: I | 1245526 | LT629780.1 | SDU39007.1 | 8.90E-23 | 84.9 | 75.701 | 107 | 1.32E-50 |
| *Pseudomonas allokribbensis* strain IzPS23 chromosome, complete genome | 2774460 | NZ_CP062252.1 | WP_096822646.1 | 1.10E-21 | 81.4 | 75.61 | 123 | 6.04E-61 |
| *Pseudomonas orientalis* strain LMG 23660 chromosome I | 76758 | NZ_LT629782.1 | WP_057721801.1 | 1.80E-21 | 80.7 | 75.61 | 123 | 1.17E-60 |
| *Pseudomonas yamanorum* strain LMG 27247 genome assembly, chromosome: I | 515393 | LT629793.1 | SDT95385.1 | 3.10E-22 | 83.2 | 74.797 | 123 | 3.65E-61 |
| *Pseudomonas tritici* strain SWRI145 chromosome, complete genome | 2745518 | NZ_CP077084.1 | WP_065872076.1 | 6.50E-22 | 82.1 | 74.797 | 123 | 3.77E-60 |
| *Pseudomonas lurida* strain LMG 21995 Ga0070647_11, whole genome shotgun sequence | 244566 | NZ_PDJB01000001.1 | WP_003232122.1 | 2.20E-21 | 80.4 | 74.797 | 123 | 9.16E-60 |
| *Pseudomonas prosekii* strain LMG 26867 genome assembly, chromosome: I | 1148509 | LT629762.1 | SDR94911.1 | 3.40E-22 | 83 | 73.984 | 123 | 6.11E-61 |
| *Pseudomonas rhodesiae* strain LMG 17764 chromosome I | 76760 | NZ_LT629801.1 | WP_034138572.1 | 2.00E-21 | 80.6 | 73.984 | 123 | 4.49E-59 |
| *Pseudomonas fluorescens* strain ATCC 13525 chromosome I | 294 | NZ_LT907842.1 | WP_034098661.1 | 1.40E-21 | 81.1 | 73.984 | 123 | 3.69E-59 |
| *Pseudomonas tolaasii* strain CCUG 23369 contig_0000001, whole genome shotgun sequence | 29442 | NZ_VZPT01000001.1 | WP_016969266.1 | 1.10E-21 | 81.4 | 73.984 | 123 | 3.45E-59 |
| *Pseudomonas cavernicola* strain K1S02-6 Segkk0, whole genome shotgun sequence | 2320866 | NZ_QYUR01000001.1 | WP_119952152.1 | 2.70E-23 | 86.6 | 72.581 | 124 | 6.97E-61 |
| *Pseudomonas cavernae* strain K2W31S-8 chromosome, complete genome | 2320867 | NZ_CP032419.1 | WP_119893713.1 | 1.40E-22 | 84.3 | 72.358 | 123 | 4.44E-60 |
| *Pseudomonas vanderleydeniana* strain RW8P3 chromosome, complete genome | 2745495 | NZ_CP077093.1 | WP_186684617.1 | 8.30E-22 | 81.8 | 71.545 | 123 | 6.89E-58 |
| *Pseudomonas quercus* strain hsmgli-8 Scaffold1, whole genome shotgun sequence | 2722792 | NZ_JAAVJI010000001.1 | WP_168080333.1 | 7.90E-23 | 85.1 | 69.919 | 123 | 1.96E-51 |
| *Pseudomonas asplenii* strain ATCC 23835 chromosome I | 53407 | NZ_LT629777.1 | WP_090207135.1 | 5.70E-22 | 82.3 | 69.919 | 123 | 1.48E-56 |
| *Pseudomonas baltica* strain MBT-2 PS60_contig_1, whole genome shotgun sequence | 2762576 | NZ_JACMYH010000001.1 | WP_197975812.1 | 7.80E-22 | 81.9 | 69.912 | 113 | 1.24E-52 |
| *Pseudomonas rhizosphaerae* strain DSM 16299 chromosome, complete genome | 216142 | NZ_CP009533.1 | WP_043190046.1 | 4.60E-23 | 85.8 | 69.106 | 123 | 5.71E-56 |
| *Stutzerimonas balearica* DSM 6083 strain DSM6083 (=SP1402) chromosome, complete genome | 1123016 | NZ_CP007511.1 | WP_041105557.1 | 6.70E-24 | 88.5 | 63.303 | 109 | 4.17E-41 |
| *Stutzerimonas stutzeri*, complete sequence | 316 | NC_015740.1 | WP_011913416.1 | 1.40E-22 | 84.3 | 62.963 | 108 | 2.07E-41 |
| *Stutzerimonas kirkiae* strain P4C NODE_10_length_118934_cov_12.356519, whole genome shotgun sequence | 2211392 | NZ_QJUO01000010.1 | WP_131184110.1 | 1.60E-21 | 80.8 | 60.976 | 123 | 7.88E-47 |
| *Pseudomonas pohangensis* strain DSM 17875 chromosome I | 364197 | NZ_LT629785.1 | WP_090193582.1 | 4.80E-25 | 92.2 | 57.724 | 123 | 1.53E-35 |
| *Pseudomonas silesiensis* strain A3 chromosome, complete genome | 1853130 | NZ_CP014870.1 | WP_064676368.1 | 3.30E-22 | 83.1 | 46.988 | 83 | 1.34E-12 |
| *Pseudomonas arsenicoxydans* strain CECT 7543 chromosome I | 702115 | NZ_LT629705.1 | WP_090185466.1 | 1.80E-21 | 80.7 | 46.988 | 83 | 4.40E-12 |
| *Pseudomonas moorei* strain CCUG 53114 contig_0000001, whole genome shotgun sequence | 395599 | NZ_VZPP01000001.1 | WP_090322306.1 | 3.90E-22 | 82.8 | 46.988 | 83 | 9.12E-13 |
| *Pseudomonas zeae* strain OE 48.2 chromosome, complete genome | 2745510 | NZ_CP077090.1 | WP_186621073.1 | 7.80E-22 | 81.9 | 45.783 | 83 | 5.51E-12 |
| *Pseudomonas knackmussii* B13 chromosome I, complete sequence | 1301098 | NZ_HG322950.1 | WP_043254542.1 | 6.30E-22 | 82.2 | 40.171 | 117 | 8.10E-12 |
| *Pseudomonas frederiksbergensis* strain AS1 chromosome, complete genome | 104087 | NZ_CP018319.1 | WP_076030497.1 | 5.50E-22 | 82.3 | 39.496 | 119 | 4.00E-13 |
| Pseudomonas taetrolens strain NCTC10697 chromosome 1, complete sequence | 47884 | NZ_LS483370.1 | WP_048382053.1 | 1.00E-19 | 75.1 | 39.241 | 79 | 3.85E-06 |
| Pseudomonas deceptionensis strain LMG 25555, whole genome shotgun sequence | 882211 | NZ_FNUD01000002.1 | WP_048360965.1 | 6.10E-20 | 75.8 | 38.667 | 75 | 1.01E-04 |
| Pseudomonas allokribbensis strain IzPS23 chromosome, complete genome | 2774460 | NZ_CP062252.1 | WP_192560681.1 | 3.20E-22 | 83.1 | 38.655 | 119 | 9.03E-13 |
| Pseudomonas tensinigenes strain ZA 5.3 chromosome, complete genome | 2745511 | NZ_CP077089.1 | WP_055135309.1 | 4.60E-22 | 82.6 | 38.655 | 119 | 9.22E-13 |
| Pseudomonas kielensis strain MBT-1 200706PS37_contig_1, whole genome shotgun sequence | 2762577 | NZ_JACMYG010000001.1 | WP_185817788.1 | 6.40E-22 | 82.1 | 38.655 | 119 | 6.48E-13 |
| Pseudomonas arsenicoxydans strain CECT 7543 chromosome I | 702115 | NZ_LT629705.1 | WP_090184027.1 | 8.90E-22 | 81.7 | 38.655 | 119 | 2.17E-12 |
| Pseudomonas marginalis strain DSM 18529 10_261255_49.9649, whole genome shotgun sequence | 298 | NZ_VFER01000010.1 | WP_146477815.1 | 1.20E-21 | 81.3 | 38.655 | 119 | 7.69E-13 |
| Pseudomonas xantholysinigenes strain RW9S1A chromosome, complete genome | 2745490 | NZ_CP077095.1 | WP_186659621.1 | 5.90E-22 | 82.3 | 38.136 | 118 | 2.98E-11 |
| Pseudomonas muyukensis strain COW39 chromosome, complete genome | 2842357 | NZ_CP077073.1 | WP_217853825.1 | 4.10E-21 | 79.5 | 38.017 | 121 | 6.30E-11 |
| Pseudomonas versuta strain L10.10 chromosome, complete genome | 1788301 | NZ_CP012676.1 | WP_060693172.1 | 1.40E-19 | 74.7 | 37.975 | 79 | 2.74E-05 |
| Pseudomonas entomophila L48, complete sequence | 384676 | NC_008027.1 | WP_011534578.1 | 5.60E-21 | 79.1 | 37.903 | 124 | 8.73E-13 |
| Pseudomonas salmasensis strain SWRI126 chromosome, complete genome | 2745514 | NZ_CP077083.1 | WP_017337059.1 | 1.10E-21 | 81.4 | 37.815 | 119 | 2.22E-12 |
| Pseudomonas arsenicoxydans strain CECT 7543 chromosome I | 702115 | NZ_LT629705.1 | WP_090181980.1 | 5.20E-22 | 82.4 | 37.815 | 119 | 3.44E-13 |
| Pseudomonas arsenicoxydans strain CECT 7543 chromosome I | 702115 | NZ_LT629705.1 | WP_090182443.1 | 9.20E-22 | 81.6 | 37.815 | 119 | 6.07E-12 |
| Pseudomonas arsenicoxydans strain CECT 7543 chromosome I | 702115 | NZ_LT629705.1 | WP_090185437.1 | 7.80E-07 | 33.8 | 32.5 | 80 | 1.50E+00 |
| Pseudomonas versuta strain L10.10 chromosome, complete genome | 1788301 | NZ_CP012676.1 | WP_060693170.1 | 2.30E-16 | 64.3 | 31.452 | 124 | 6.00E-03 |
| Pseudomonas taetrolens strain NCTC10697 chromosome 1, complete sequence | 47884 | NZ_LS483370.1 | WP_048382054.1 | 1.70E-16 | 64.7 | 31.452 | 124 | 1.00E-03 |
| Pseudomonas deceptionensis strain LMG 25555, whole genome shotgun sequence | 882211 | NZ_FNUD01000002.1 | WP_048360966.1 | 1.60E-16 | 64.8 | 30.645 | 124 | 5.00E-03 |
| Pseudomonas putida NBRC 14164 DNA, complete genome | 1211579 | AP013070.1 | BAN53605.1 | 1.40E-23 | 87.5 | 100 | 124 | 2.44E-82 |

**References**

Lalucat J, Gomila M, Mulet M, Zaruma A, García-Valdés E. 2022. Past, present and future of the boundaries of the *Pseudomonas* genus: Proposal of *Stutzerimonas* gen. *Nov Syst Appl Microbiol* 45:126289. doi: 10.1016/j.syapm.2021.126289. Epub 2021 Dec 6. PMID: 34920232.
